# Evaluating Performance of EEG Data-Driven Machine Learning for Traumatic Brain Injury Classification

**DOI:** 10.1109/TBME.2021.3062502

**Published:** 2021-10-19

**Authors:** Nicolas Vivaldi, Michael Caiola, Krystyna Solarana, Meijun Ye

**Affiliations:** Office of Science and Engineering Laboratories, Center for Devices and Radiological Health, U.S. Food and Drug Administration, USA. He is now with the MathWorks, USA; Office of Science and Engineering Laboratories, Center for Devices and Radiological Health, U.S. Food and Drug Administration, USA.; Office of Science and Engineering Laboratories, Center for Devices and Radiological Health, U.S. Food and Drug Administration, USA.; Office of Science and Engineering Laboratories, Center for Devices and Radiological Health, U.S. Food and Drug Administration, Silver Spring, MD 20993 USA

**Keywords:** EEG database, machine learning (ML), traumatic brain injury (TBI), stroke

## Abstract

**Objectives:**

Big data analytics can potentially benefit the assessment and management of complex neurological conditions by extracting information that is difficult to identify manually. In this study, we evaluated the performance of commonly used supervised machine learning algorithms in the classification of patients with traumatic brain injury (TBI) history from those with stroke history and/or normal EEG.

**Methods:**

Support vector machine (SVM) and K-nearest neighbors (KNN) models were generated with a diverse feature set from Temple EEG Corpus for both two-class classification of patients with TBI history from normal subjects and three-class classification of TBI, stroke and normal subjects.

**Results:**

For two-class classification, an accuracy of 0.94 was achieved in 10-fold cross validation (CV), and 0.76 in independent validation (IV). For three-class classification, 0.85 and 0.71 accuracy were reached in CV and IV respectively. Overall, linear discriminant analysis (LDA) feature selection and SVM models consistently performed well in both CV and IV and for both two-class and three-class classification. Compared to normal control, both TBI and stroke patients showed an overall reduction in coherence and relative PSD in delta frequency, and an increase in higher frequency (alpha, mu, beta and gamma) power. But stroke patients showed a greater degree of change and had additional global decrease in theta power.

**Conclusions:**

Our study suggests that EEG data-driven machine learning can be a useful tool for TBI classification.

**Significance:**

Our study provides preliminary evidence that EEG ML algorithm can potentially provide specificity to separate different neurological conditions.

## I. Introduction

Traumatic brain injury (TBI) presents a significant challenge to civilian and military medicine. According to the Centers for Disease Control and Prevention (CDC), an estimated 2.5 million people sustain a TBI annually, contributing to a third of all injury-related deaths in the United States. Given the high societal and economic costs of untreated TBI, it is recognized as a significant military and public health concern. Currently, neurological Glasgow Coma Scale (GCS) is a clinical index universally used to classify TBI as mild, moderate or severe. CT scan is used to detect structural brain lesions. Though useful in the clinical management of TBI, these methods do not provide enough sensitivity to detect mild TBI and monitor the progression of TBI at different severities. Therefore, efforts are ongoing to seek for alternative clinical assessment tools for TBI, including body-fluid analysis, advanced imaging modalities (i.e., diffuse tensor imaging [DTI], positron emission tomography [PET]) and neurophysiological signals (i.e., eye movement and electroencephalography [EEG]).

Among all the modalities, EEG has advantages of being non-invasive, easy-to-use, portable and cost effective. However, when applied to TBI research, EEG yields mixed results in the literature. Views on the clinical significance of EEG in TBI assessment are historically controversial [1]–[4]. Studies have shown significant differences in EEG-based power spectra data between mild TBI and normal groups [5], [6], while other studies report no such distinction [7]. Researchers have also evaluated post-TBI changes in connectivity [8], [9] and entropy [10], [11]. Abnormal electrophysiological signals were observed to occur without structural and biochemical changes following neural disruptive interventions, or even in the lack of apparent neurocognitive abnormality [10], [12], suggesting that EEG has the potential to be a sensitive indicator of neuropathology. However, how specific these changes are to TBI is questionable.

With the advancement of computational analytical technologies, the clinical utility of EEG signals may be propelled significantly. Health-related research has benefited from data-mining machine learning (ML) techniques built on the increasingly available wealth of information provided by large scale repositories [13]. Due to the inherent complexity of TBI, including the absence of consensus on biomarkers, underlying relationships between data, and patient-to-patient variability, big data analytics have the potential to make determinations about population characteristics that would otherwise be too difficult or impossible to manually identify [14], [15]. While clinicians receive extensive training to interpret EEG signals, advances in ML and deep learning may enable data-driven computational systems to emulate and even improve this process. Particularly, multiple kinds of temporal and spectral analyses can be performed on EEG recordings across multiple channels, generating feature sets that are well suited for ML applications. In addition, due to its long history of use in neurological conditions, multiple EEG databases are already formed and are publicly available. This provides a platform to evaluate the feasibility of the implementation of ML to the investigation of TBI assessment. Multivariate EEG data has previously been shown to be effective in classifying acute TBI patients with positive CT scans [16]. However, more study is necessary for broader application among groups with different stage and severity of TBI as well as different demographics.

Here, we evaluated the performance of multiple commonly used ML algorithms in the classification of patients with TBI history from normal subjects with a diverse feature set composed of demographic information, power spectral density, channel-to-channel coherence, phase-amplitude coupling, and spectral entropy, from Temple EEG Corpus. In addition, we further assessed the accuracy of algorithms in the classification of TBI, stroke, and normal patients to determine the specificity.

## II. Methods

### A. EEG Data

Raw EEG data was obtained from the Temple University Hospital EEG Corpus repository (v1.1.0), the world’s largest clinical EEG database [17]. Subjects were identified through patient records associated with each EEG file (.EDF format). Records were parsed using Python scripts as described in paper [18] with key words listed in Sup Fig. 1 (available at - https://github.com/dbp-osel/qEEG-consistency), further curated by a custom MATLAB (MathWorks, Version 2019b, Natick, MA, USA) script, then verified by manually inspecting the content of each automatically selected record to assure their compliance with inclusion and exclusion criteria listed below.

#### 1) Inclusion Criteria:

##### TBI group:

1) ages 1–85 and 2) medical record includes a diagnosis of TBI or concussion.

##### Stroke group:

1) ages 1–85 and 2) medical record of a stroke diagnosis.

##### Normal group:

1) ages 1–85 and 2) clinicians’ notes indicated that the EEG was within normal ranges for the subject’s demographic group.

#### 2) Exclusion Criteria:

##### TBI group:

Documented history of epilepsy, seizure, tremors, or other neurological conditions other than TBI within the clinicians’ note record.

##### Stroke group:

Documented history of epilepsy, seizure, tremors, or other neurological conditions other than stroke within the clinicians’ note record.

##### Normal group:

Documented history of epilepsy, seizure, tremors, or other neurological conditions within the clinicians’ note record.

All patient data in the database were de-identified. Therefore, this study did not constitute human subjects research, and was exempted from Food and Drug Administration institutional review board review. A total of 13550 subjects were analyzed and processed according to the description in Sup. Fig. 1. The final dataset for training machine learning models included 292 subjects with 79 normal labels, 98 TBI labels, and 115 stroke labels. A total of 26 normal labeled, 44 TBI labeled, and 50 stroke labeled subjects’ data were randomly reserved for use as an independent validation (IV) set. There was no overlap between any of the cohorts.

It needs to be acknowledged that in the Temple database, information on the occurrence time of TBI/stroke was often unavailable, nor were the severity and cause of the injury consistently reported. Therefore, the composition of the patient was heterogenous in both diseases’ groups.

### B. EEG Data Preprocessing

**Fig. 1.**
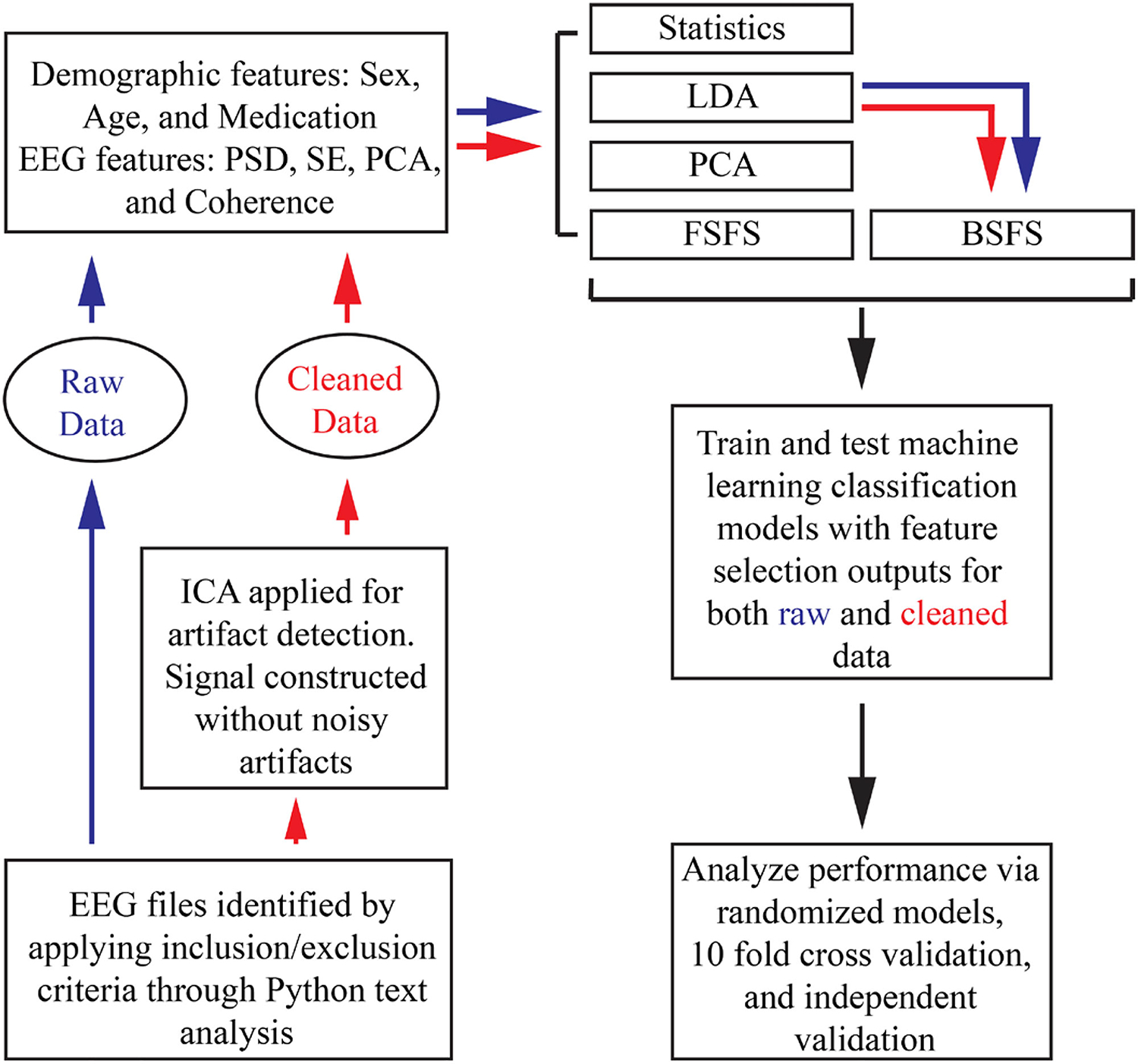
Flowchart describing data processing and model training. (ICA: independent component analysis, PSD: power spectral density, SE: spectral entropy, PAC: phase-amplitude coupling, LDA: linear discriminant analysis, PCA: principal component analysis, FSFS: forward sequential feature selection, BSFS: backwards sequential feature selection).

Fig. 1 depicts the data processing, feature generation, feature selection, model training and validation flowchart.

EEG signals from different subjects were first normalized so that individual records conform with one another in terms of channels used, length of time of the recording, consistent epoching, and sampling frequency (fs). Specifically, for each subject, 3 minutes of awake, resting-state, stimuli-free EEG recordings were included (excluding the first minute of the recording). EEG data were further pre-processed using MATLAB and *eeglab* (v.2020.0) [19]. Signals (fs = 250 Hz) from 19 common channels in ten-twenty standard arrangement (FP1, FP2, F3, F4, C3, C4, P3, P4, O1, O2, F7, F8, T7, T8, P7, P8, FZ, CZ, PZ) were filtered using *pop_eegfiltnew()* with cutoff frequency passband 1–100 Hz. Conventionally, channels with poor signal quality are dropped from EEG analysis. However, no channels were rejected due to the need to keep the feature vector for each subject consistent, thereby making the assumption that the collective feature space across all subjects would be robust to outliers. Filtered signals were re-referenced via *pop_reref()* to remove background noise by subtracting the average amplitude across all channels at each discrete time point from each channel’s signal individually. The resulting filtered and re-referenced signals were referred to as raw data.

Artifact rejection was performed in order to evaluate the performance of TBI classifiers using both raw and cleaned data. Independent component analysis (ICA) is a proven computational technique for EEG artifact detection [20] and was applied using the FastICA (v.2.5) package for MATLAB [21]. Input EEG channel data are separated into each independent component (IC) that are linearly mixed in the original signal. This occurs through singular value decomposition of the EEG data. ICLabel (v.1.2.6), a plugin for *eeglab*, was then used to classify each channel’s ICs by their probabilistic source. ICLabel is a classification tool that was trained on thousands of known, labeled signals including EEG, EMG, EOG, etc. Each IC was labeled as brain, muscle, eye, heart, line noise, channel noise, or other according to the highest probabilistic source. ICs with non-brain function sources such as eye movements were excluded from signal reconstruction. Artifact rejected data were referred to as cleaned data.

### C. Feature Generation

Raw and cleaned signals were processed using spectral analysis techniques in order to generate a descriptive vector of quantitative features describing each subject’s EEG recording. The spectral features calculated were: phase-amplitude coupling (PAC) [22], absolute and relative power spectral density (PSD) within frequency bands, spectral entropy (SE), and inter-channel cross coherence (Coh) resulting in 1330 EEG features for both raw and cleaned data. For the purpose of feature generation, the frequency bands used were defined as 1 – 4 Hz (delta), 4 – 8 Hz (theta), 8 – 12 Hz (alpha), 12 – 16 Hz (mu), 16 – 20 Hz (beta), and 25 – 40 Hz (gamma).

Absolute PSD was calculated in each frequency band using the *bandpower()* MATLAB function. Relative PSD was calculated by dividing absolute PSD in each frequency band by PSD between 1 and 100 Hz. Similarly, coherence was calculated using MATLAB’s *mscohere()* with 30 second non-overlapping epoch windows. Spectral entropy (*H*) was calculated with custom written MATLAB code using the equation:

$$H=-\sum_{m=1}^{N}\frac{S(m)}{\sum_{i}S(i)}log_{2}\left(\frac{S(m)}{\sum_{i}S(i)}\right)$$

Where *S(m)* is the power spectrum of the input (channel-wise) signal and *N* is the total number of data points. Phase-amplitude coupling was calculated following the method presented in [22] which determines the modulation index (*MI*) between phase bins and amplitudes via Kullback-Leibler distance (*D*_*KL*_):

$$MI=\frac{D_{KL}(P,U)}{log(N)}$$

Where *P* is the amplitude distribution among *N* = 18 phase bins (−180° to 180°), and U is the uniform distribution. Pairings of amplitude and phase were tested between bands alpha and gamma, theta and gamma, and theta and alpha.

### D. Feature Selection

Dimensionality reduction was performed via five methods: conventional statistics, Principal Component Analysis (PCA), Linear Discriminant Analysis (LDA), Forward Sequential Feature Selection (FSFS), and Backwards Sequential Feature Selection (BSFS). Apart from the statistics method, all features were standardized to account for large variations in data ranges.

#### 1) Statistics:

One sample Kolmogorov-Smirnov test (K-S test) was used to determine normal distribution of the data at the 5% significance level. None of the features were normally distributed, therefore, Wilcoxon rank sum testing was implemented to analyze differences. When used with three classes, One-way ANOVA and post-hoc Tukey test was used instead of the Wilcoxon rank sum test. False discovery rate was set at 0.05 and significant p-values were calculated for multiple comparison correction [23 ]. First, all ***m*** p-values were ranked ***p***_1_, . . . , ***p*_*m*_** with ***p***_1_ being the smallest. Then we denote ***p***_1_, . . . , ***p***_***k***_ significant for the largest ***k*** that satisfies:

$$p_{k}\leq \frac{0.05k}{m}$$

Only Features With P-Value With Rank 1, . . . , *k* Were Used to Train Classifiers.

#### 2) LDA:

Although LDA itself can be used as a classifier model, here it is used to identify which subset of the original features best separate the classes. LDA was optimized by selecting the best delta and gamma values over a 50-step grid search. Features with *δ* coefficient values below the cutoff threshold were eliminated from the data later passed on to the models for training. In this study, the threshold was set to the mean of the *δ* values plus one standard deviation.

#### 3) FSFS:

For this analysis the criterion value was set to the minimum mean misclassification error over 10-fold cross-validation (CV) of linear discriminant models after 50 Monte Carlo repetitions.

#### 4) BSFS:

For this analysis the criterion value was set to the minimum mean misclassification error over 10-fold cross-validation of linear discriminant models after 50 Monte Carlo repetitions. This technique selected features backwards starting with the previously selected LDA features instead of the original 1330 feature set.

#### 5) PCA:

PCA was selected as a dimensionality reduction technique due to its advantage of not eliminating potentially useful information by dropping features. PCA was applied to both the raw and cleaned feature sets specified to account for ≥ 95% of the total variation within the space.

### E. Model Training

Feature vectors identified by statistics, PCA, LDA, FSFS, and BSFS were fed into Support Vector Machine (SVM) models using six different kernels and K-Nearest Neighbors (KNN) classifiers using six definitions for training. SVM used three polynomial kernels (linear, quadratic, and cubic) and three Gaussian kernels. Gaussian kernel scale was determined as 4∗*sqrt*(N), where N = number of features, *sqrt*(N), and *sqrt*(N)/4 for models named coarse, medium, and fine, respectively. Three KNN models were trained based on Euclidean distance for K = 1, 10, and 100, also denoted coarse, medium, and fine, respectively. Two more KNN models were trained with K = 10 using cosine and cubic distances. The last model trained was KNN with K = 10 where neighbors were weighted by the squared inverse of their Euclidean distance. For both raw and clean data using five selection methods and 12 total models, 120 total models were trained and tested. An additional 120 models were trained and tested as above, intentionally excluding demographic features. These model variations were trained on two-class data (normal vs TBI) and three-class data (normal vs TBI vs stroke), for a total of 480 models.

### F. Model Validation

Performance of classifiers were validated with 10-fold cross-validation (CV), label randomization, and IV data set (Table I). Model accuracy, F1 score, sensitivity, and specificity were recorded to assess the performance of each model to classify TBI and normal data. For three-class classifiers to classify TBI, normal, and stroke, macro and weighted variants of precision/sensitivity/F1 scores were calculated.

In order to assess a baseline metric of performance, group labels (TBI, normal and stroke) were randomly assigned to the feature vectors and each randomized set was trained and tested following the same methods used for true labeled data. Both true and randomly labeled data were trained using 10-Fold CV partitions 1000 times in order to generate distributions of cumulative prediction accuracies over each fold. In addition, classifiers generated with the full training data set were further evaluated by predicting classifications of an independent test data set with 120 true labeled subjects, which were withheld from the training data set (See Table I for demographic information of each data set).

To further determine validation, a model was considered a “success” if its validation score was at least above that of the Zero Rule (ZeroR) benchmark. The ZeroR benchmark is calculated as the accuracy of a model that predicts the largest class no matter the input. For the normal vs TBI models, the ZeroR benchmarks were at 55.37% for the CV tests and 62.86% for the IV tests. For the three-class models, ZeroR was at 39.7% and 41.67% for the CV and IV, respectively.

### G. Statistical Analysis

Statistical analysis was performed to provide baseline measure of the differences between the TBI, stroke and normal classes on the individual features, and to compare performance of models. One sample Kolmogorov-Smirnov test (K-S test) was used to determine normal distribution of the data at the 5% significance level. If data was not normal distributed, Wilcoxon rank sum testing, or two sample K-S test, or signed rank test, were implemented to analyze differences. When data was normally distributed, Student t-test was used. Where it is applicable, positive false discovery rate was calculated for multiple comparison correction. For categorical sex data, Chi square test was used. When comparing the five feature selection methods, One Way ANOVA and post-hoc Tukey test was used. All statistical data are expressed as mean ± std.

## III. Results

### A. Performance of Individual Two-Class Algorithms

All 1330 EEG features and 3 demographic features (sex, age, and medication) of normal and TBI cohorts were put through statistical analysis, LDA, FSFS, BSFS, and PCA for feature selection. Sex is set as categorical data. Medication is set as the number of unique medications prescribed to each subject. Drug interaction was not investigated in this study.

#### 1) Models Trained With Features Selected By Statistics:

In statistical analysis, rank sum and false discovery rate analyses identified 98 features out of the 1333 in the raw set (~7.35%) and 82 in the clean set (~6.15%) that were significantly different between TBI and normal subjects, including sex. When comparing the performance of models trained with truly labeled data and randomly labeled data with 10-fold CV, all models trained with truly labeled performed significantly better than randomly labeled data with the only exception of SVM fine Gaussian at 10^−10^ significance level (SL) (Sup. Fig. 2) (two sample K-S test, p<10^−10^, 1000 iterations). Models trained with truly labeled data had an average accuracy of 0.68 ± 0.05 with median at 0.68. In addition to 10-fold CV, we evaluated the performance of models with an independent data set which were withheld from training. In general, the performance of models to predict the classification of independent data set was better than 10-fold CV (Sup. Fig. 2). The average accuracy of the 24 models was 0.70 ± 0.05 with median at 0.71.

#### 2) Models Trained With Features Selected By LDA:

**Fig. 2.**
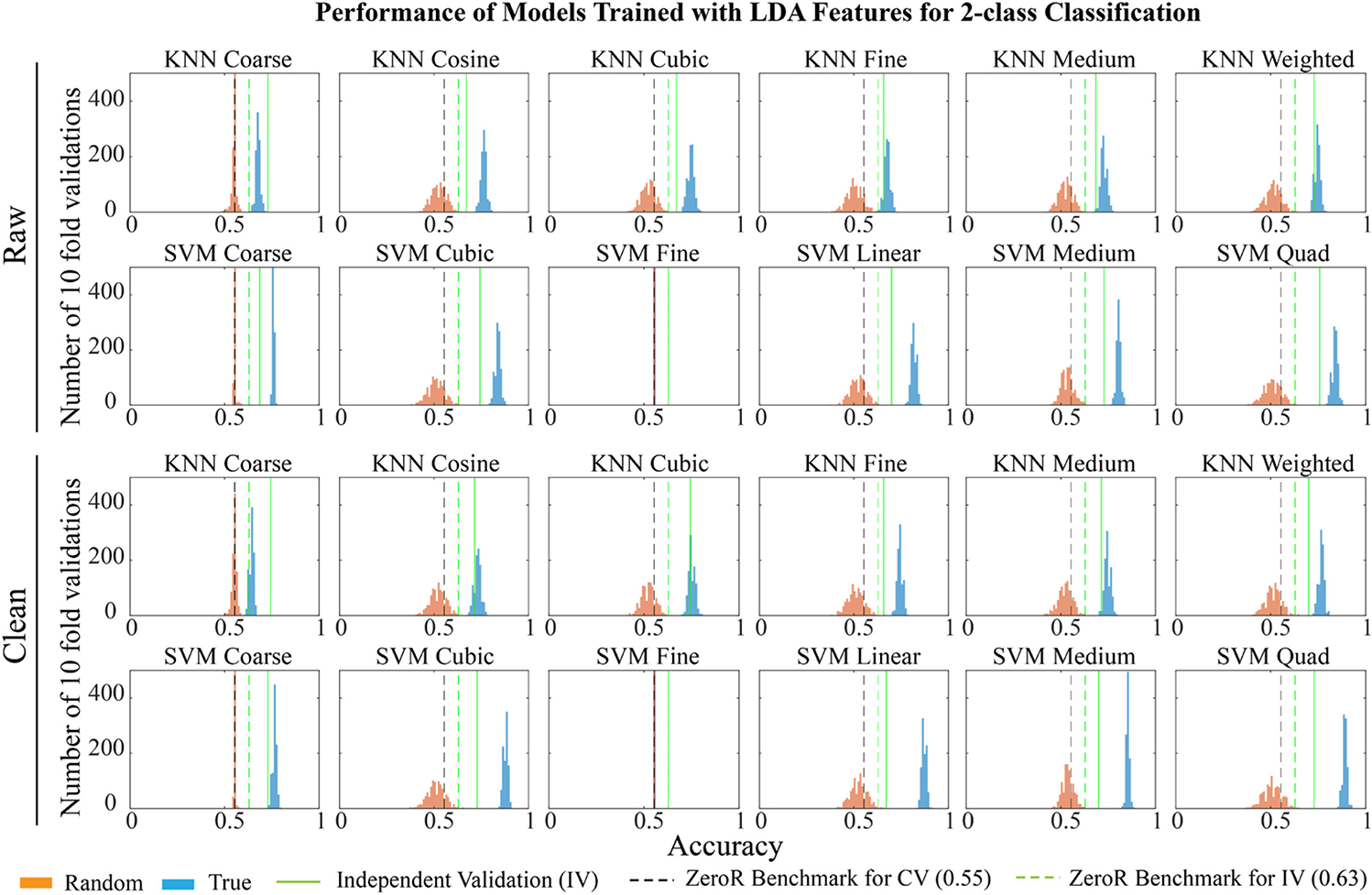
Performance of models trained with features selected by linear discriminant analysis (LDA). The figure shows distribution of accuracies for 1000 iterations of training using randomly labeled data (orange), true labeled data (blue), and independent dataset (green line) for models based on features selected by LDA. Black and green dotted lines show ZeroR benchmarks for cross-validation (CV) and independent validation (IV) respectively. All models trained with true labeled data performed significantly better than randomly labeled data at 10^−10^ confidence interval in 10-fold CV, except for SVM fine Gaussian models (two sample K-S test). (SVM: support vector machine, KNN: K-nearest neighbors).

With a threshold of the mean of the *δ* values plus one standard deviation, LDA selected 208 features from raw data features, and 224 from clean features, including two demographic features, sex and age. 1000 iterations of 10-fold CV showed that 22 out of 24 models trained with truly labeled data performed significantly better than those trained with randomly labeled data (Fig. 2) (n = 1000, p < 10^−10^, two sample K-S test). Like models trained with statistically selected features, SVM algorithm with fine Gaussian kernel could not distinguish TBI and normal subjects at all. When classifying the independent data set, the 24 models showed an average accuracy of 0.76 ± 0.09 with median at 0.75 (Fig. 2), which is slightly better than the accuracy calculated with 10-fold CV, 0.70 ± 0.04 with median at 0.70.

#### 3) Models Trained With Features Selected By FSFS:

With the criterion described in Methods, FSFS selected sex and an additional 8 EEG features from raw data, and 12 from clean data. All 24 models trained with truly labeled data performed significantly better than those trained with randomly labeled data when evaluated with 1000 iterations of 10-fold CV at 10^−10^ SL with a mean accuracy of 0.71 ± 0.06 with median at 0.73 (Sup. Fig. 3) (two sample K-S test). When these models were evaluated by the independent data set, they showed an average accuracy of 0.67 ± 0.05 with median at 0.67 (Sup. Fig. 3).

#### 4) Models Trained With Features Selected By BSFS:

Working backwards from the features selected by LDA, BSFS selected sex, age, and an additional 179 EEG features from raw data and 216 from clean data. Similar to the LDA models, all models trained with truly labeled data, with the exception of the SVM algorithm with fine Gaussian kernel, performed significantly better than those trained with randomly labeled data when evaluated with 1000 iterations of 10-fold CV at 10^−10^ SL. The mean accuracy of the truly labeled data set was almost as high as LDA at 0.75 ± 0.09 with median at 0.75 (Sup. Fig. 4) (two sample K-S test) which is not too surprising as these features were derived from the LDA feature set. However, when these models were evaluated by the independent data set, they performed especially poor with an average accuracy of 0.56 ± 0.06 with median 0.56 (Sup. Fig. 4).

#### 5) Models Trained With Principal Component (PC) Features:

When putting through all 1332 non-categorical features into PCA, 132 principal components (PCs) were necessary to reach 95% threshold using raw or clean data. Models were trained with the selected PC features and the categorical sex information. The majority models trained with true labeled data performed significantly better than randomly labeled data with p < 10^−10^ but the coarse and fine variants of SVM and KNN, as well as clean KNN cubic distance model (Sup. Fig. 5) (two sample K-S test). Additionally, 6 of the 24 models failed to have better accuracy than the Zero Rule (ZeroR) benchmark of 0.55 (black dotted line). When evaluated with 10-fold CV, the mean accuracy of these 24 models was 0.58 ± 0.05 with median at 0.57. The performance of the 24 models to classify the independent data set also performed unsatisfactorily, with a mean of 0.48 ± 0.10, a median at 0.43, and none of the models performing better than the ZeroR benchmark of 0.63 (green dotted line).

**Fig. 3.**
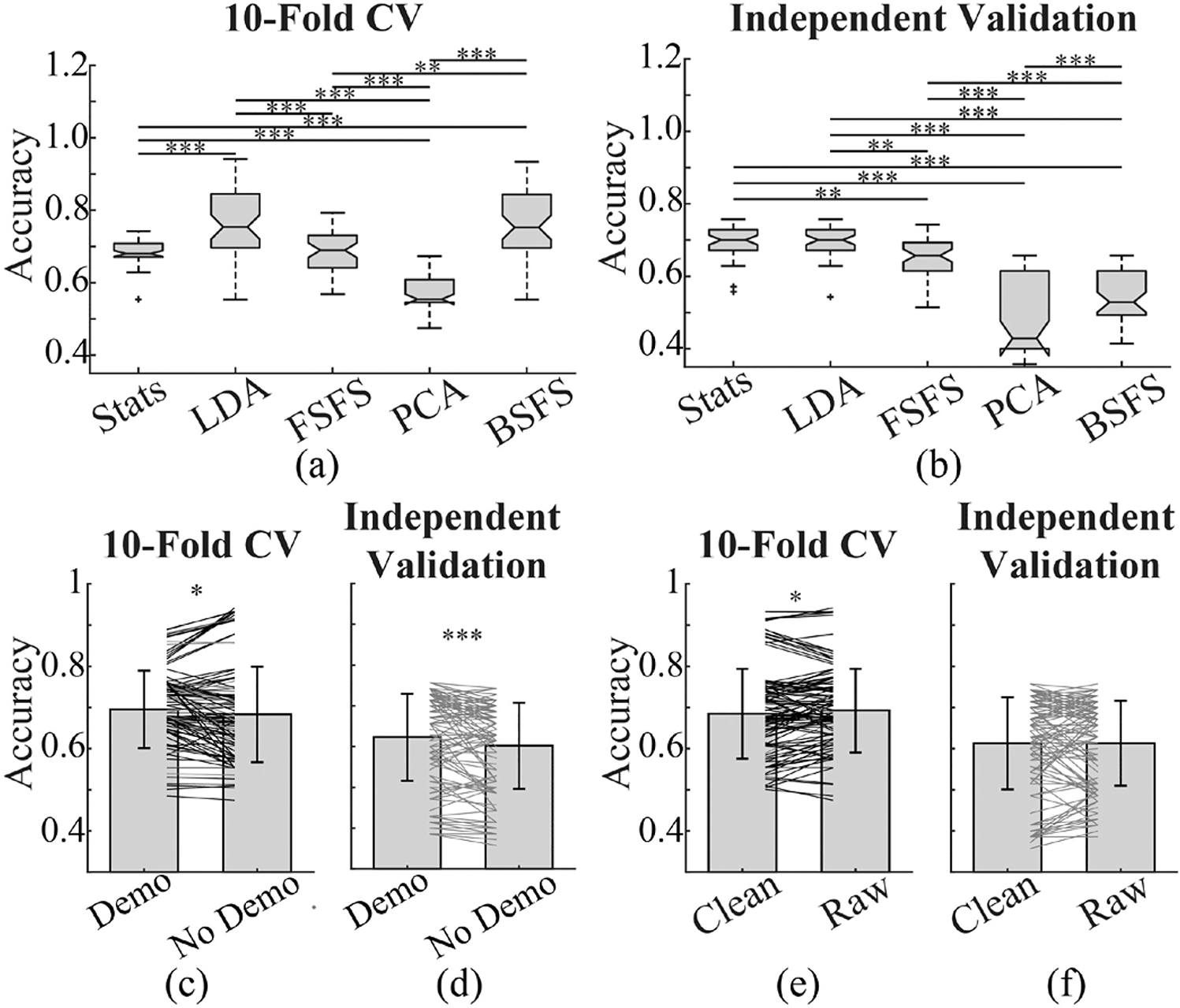
Comparison of performance of models for classifying patients with TBI history from normal subjects. (a) and (b) show boxplots of accuracy of models trained with features selected by different methods. Accuracy was evaluated with 10-fold CV and independent dataset respectively. Models trained with PCA selected features performed worst in both 10-fold CV and independent validation, while those trained with features selected by LDA performed best. Models trained with features selected by Statistics performed inferior to those with LDA in CV, however, their performance was comparable with LDA models when used to classify independent dataset. (c) and (d) compare the accuracy of models trained with input features including demographic information and those without demographic information (Demo: demographic). The majority models with demographic inputs appear to perform better than their counterparts. (e) and (f) compare the performance of models trained with features generated from artifact removed clean EEG data versus those from raw EEG. Though variability is present, most models trained with raw data performed slightly better than the corresponding clean data in CV. And performance of models from raw data was comparable with those from clean data for predicting independent dataset. Each line in (c) to (f) represents each algorithm. Dark lines in (c) and (e) indicate significant difference in two sample K-S test at 10^−10^ significance level. (∗ p < 0.05, ∗∗ p < 0.01, ∗∗∗ p < 0.001, One way ANOVA and post-hoc Tukey test in (a) and (b), Signed-rank test in (c) to (f).) (CV: cross-validation, Stats: statistics, LDA: linear discriminant analysis, FSFS: forward sequential feature selection, PCA: principal component analysis, BSFS: backwards sequential feature selection.).

We compared the performance of models with different feature selection methods (Fig. 3a and b) and found that models trained with PC features performed the poorest in both 10-fold CV and IV. Models trained with features selected by LDA performed consistently well in both CV and IV. Interestingly, models trained with features selected by statistics performed significantly worse than those from LDA in CV, however, they performed as well as those from LDA when used to classify the independent dataset. Models trained with features selected by FSFS preformed similarly to that of the statistics group in CV but slightly worse in IV. Models trained with BSFS, however, performed just as well as LDA models in CV but almost as poor as PCA models in IV.

### B. Effect of Demographic Information on the Performance of Models

To understand how demographic information affects the performance of models, we re-trained the models with sex, age, and medication information removed from input features, leaving just 1330 features before selection. Similarly, model performance was evaluated with 1000 iterations of 10-fold CV and an independent dataset. In 10-fold CV, models with demographic inputs performed consistently better than those without (Fig. 3c). Though variability is present in IV, the majority of models with demographic inputs perform significantly better than their counterparts (Fig. 3d). Including only those models that have accuracy higher than the ZeroR benchmark, we see that the IV accuracy for demographic group remains significantly higher (Sup. Fig. 6). Considering the significant difference in sex between normal and TBI groups in both training and IV data sets (Table I), this finding is not surprising.

### C. Effect of EEG Artifact Removal on the Performance of Models

In conventional quantitative EEG (qEEG) analysis, artifact removal is an inevitable step, but it is computationally and time demanding. Moreover, there is no perfect artifact removal method. Therefore, we are interested in understanding whether ML models built upon raw EEG can provide comparable performance with those on artifact-removed clean data. When comparing the performance of models trained with clean and raw EEG features, we found that the raw models performed slightly better (Fig. 3e). Interestingly, when predicting independent data, it appears there is no such difference in performance between most models trained with raw EEG compared to those trained with clean data (Fig. 3f). This remains true, when looking at just those models that performed better than the ZeroR benchmark (Sup. Fig. 6).

### D. Models With Best Performance in Classifying TBI

Though it appears that most ML models can distinguish TBI from normal subjects to some extent, demonstrated as significantly better performance than their counter models trained with randomly labeled data, their performance varies remarkably. Therefore, we identified models with accuracy higher than 0.9 in 10-fold CV and higher than 0.73 IV (Sup. Table I). In 10-fold CV, 12 models showed an accuracy higher than 0.9, all of which were SVM models based on LDA or BSFS feature selection with linear or polynomial kernels. In IV, 18 models had higher than 0.73 accuracy, but none of these were in the 12 best performers in 10-fold CV. These models were built upon features selected either by LDA, FSFS or statistics, including SVM and KNN models.

### E. Performance of Three-Class Models

To determine if the models above were actually distinguishing differences between normal and TBI EEGs instead of just normal and abnormal EEGs, we included EEGs from a third cohort of stroke patients. Features were reselected using the same previous features selection techniques including clean, raw, demographic-free and demographic variants. These newly selected features were then used to train error-correcting output codes (ECOC) models with a one vs one coding design and the same SVM and KNN variants used in the two-class case as learners. Overall, 240 models were each trained. Accuracy was calculated on 1000 iterations of 10-fold CV for both truly and randomly labeled data (Sup. Fig. 7–11) as well as an IV.

**Fig. 4.**
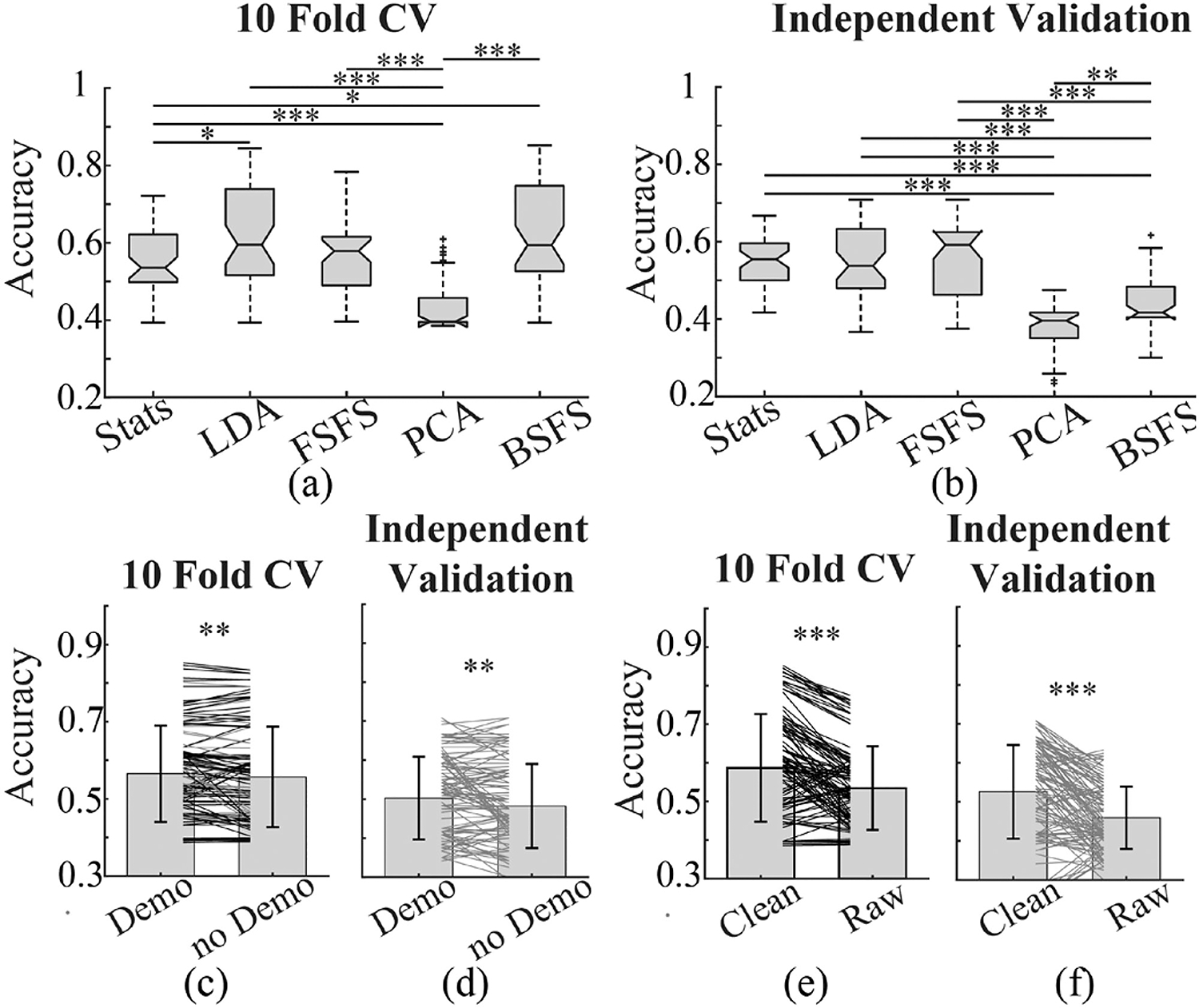
Comparison of performance of models for 3-class classification. (a) and (b) show boxplots of accuracy of models trained with features selected by different methods for classifying patients with TBI and stroke history and normal subjects. Accuracy was evaluated with 10-fold CV and independent dataset respectively. Models trained with BSFS and LDA selected features performed best in 10-fold CV. In IV, models trained with features selected by LDA, statistics, and FSFS showed comparable performance. (c) and (d) compare the accuracy of models trained with input features including demographic information and those without demographic information (Demo: demographic). The majority models with demographic inputs appear to perform better than their counterparts. (e) and (f) compare the performance of models trained with features generated from artifact removed clean EEG data versus those from raw EEG. The majority models built upon clean data performed significantly better than raw data. (∗ p < 0.05, ∗∗ p < 0.01, ∗∗∗ p < 0.001, One way ANOVA and post-hoc Tukey test in (a) and (b).) (CV: cross-validation, Stats: statistics, LDA: linear discriminant analysis, FSFS: forward sequential feature selection, PCA: principal component analysis, BSFS: backwards sequential feature selection).

There was a large variation in the CV accuracies of these models with some just over 0.85 (Sup. Table II). All models had a median accuracy above 0.5 except for those trained with features selected by PCA (Fig. 4a). For the IV: LDA, FSFS, and conventional statistics methods performed the best with PCA still being the least accurate (Fig. 4b).

Our results suggest that the features selected by LDA, FSFS, or BSFS are sufficient to produce adequate accuracy classification models between one normal group and two different disease cohorts. Ideally, with more data, this number would increase. Nevertheless, we believe this shows that our feature selection and resulting models, is detecting more than just differences between normal and abnormal EEGs. Put another way, if one were to assume the models were only detecting a difference between normal and abnormal EEGs then these models would only have an accuracy as high as

$$P(H)P(\hat{H})+P(T)P(\hat{T})+P(S)P(\hat{S})$$

with *H, T, S* representing the ground truth of a subject’s cohort, $\hat{H}$, $\hat{T}$, $\hat{S}$, representing the model’s classification of a subject’s cohort, and *P*(·) being the probability of selecting it, assuming they are chosen at random.

That is, for the case that the model always selects subjects in the Normal cohort correctly, we’d have a CV accuracy as high as

$$\left(\frac{79}{294}\right)(1)+\left(\frac{98}{294}\right)\left(\frac{98}{294}\right)+\left(\frac{117}{294}\right)\left(\frac{117}{294}\right)\approx 0.54$$

and an IV accuracy as high as

$$\left(\frac{26}{124}\right)(1)+\left(\frac{48}{124}\right)\left(\frac{48}{124}\right)+\left(\frac{50}{124}\right)\left(\frac{50}{124}\right)\approx 0.52$$

In total, 127/240 of our models have CV accuracy greater than 0.54 and 97/240 have IV accuracy greater than 0.52.

For the effect of demographic information and artifact removal on the performance of models for three-class classification, consistent with two-class models, including demographic information can increase the accuracy of models (Fig. 4c). However, opposite to the two-class models, removing EEG artifact significantly increased the accuracy of the three-class models (Fig. 4d).

**Fig. 5.**
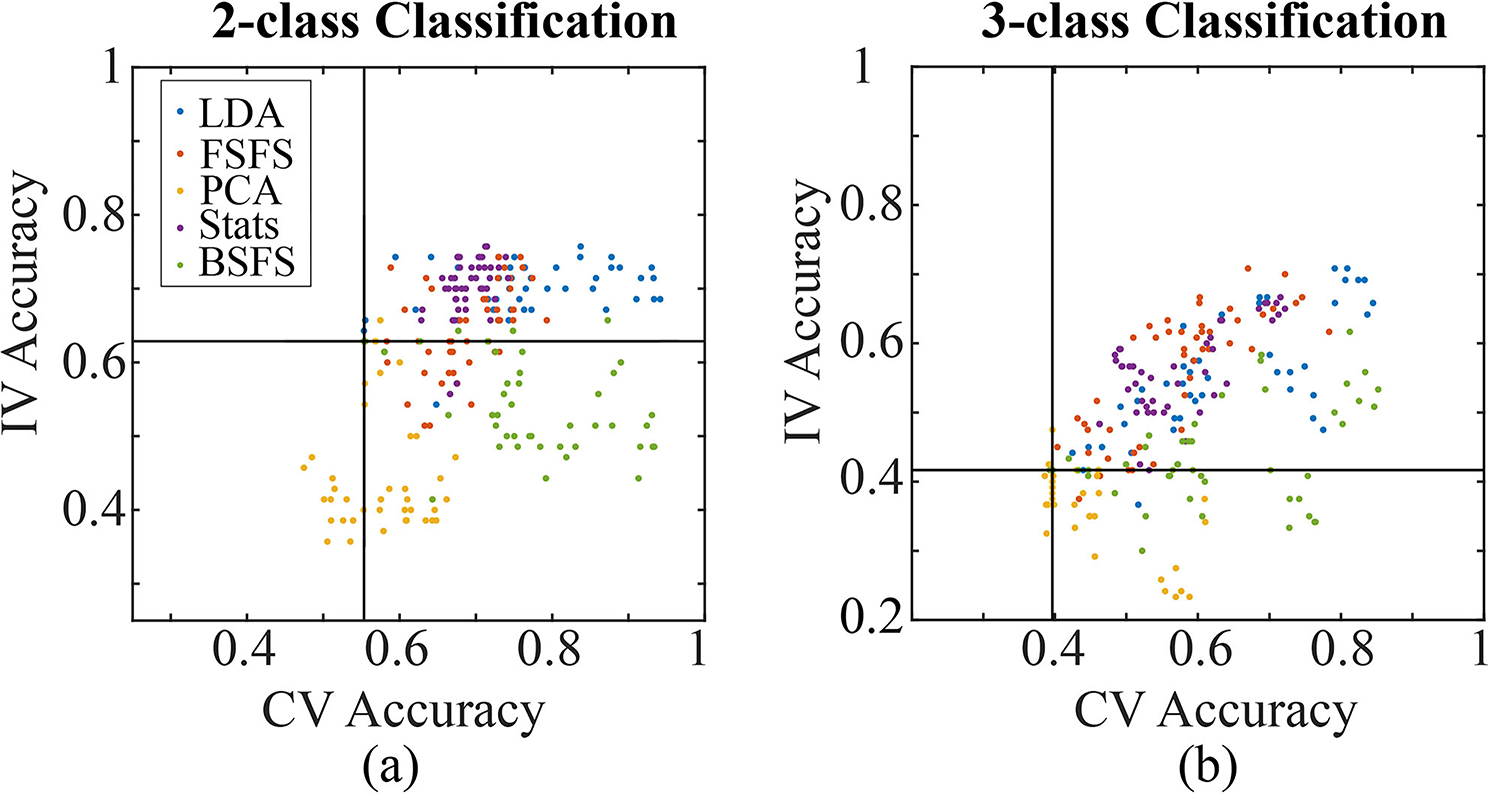
Relationship between IV and CV accuracies for two-class (a) and three-class (b) classification. The respective ZeroR benchmarks for CV and IV are shown as black lines. (CV: cross-validation, IV: independent validation, LDA: linear discriminant analysis, FSFS: forward sequential feature selection, PCA: principal component analysis, Stats: statistics, BSFS: backwards sequential feature selection.).

Taken together, our results suggest 1) LDA is the best feature selection methods among those tested for our EEG dataset (Fig. 5a and 5b), 2) SVM models perform better overall, 3) raw EEG can provide comparable performance compared with artifact-removed clean EEG in two-class classification, but significantly inferior to clean EEG in three-class classification and 4) inclusion of demographic information can slightly increase model performance for both two- and three-class models, but its role is less remarkable compared to feature selection methods and classification algorithms.

### F. Differences in qEEG Features Between Normal, TBI and Stroke Patients

**Fig. 6.**
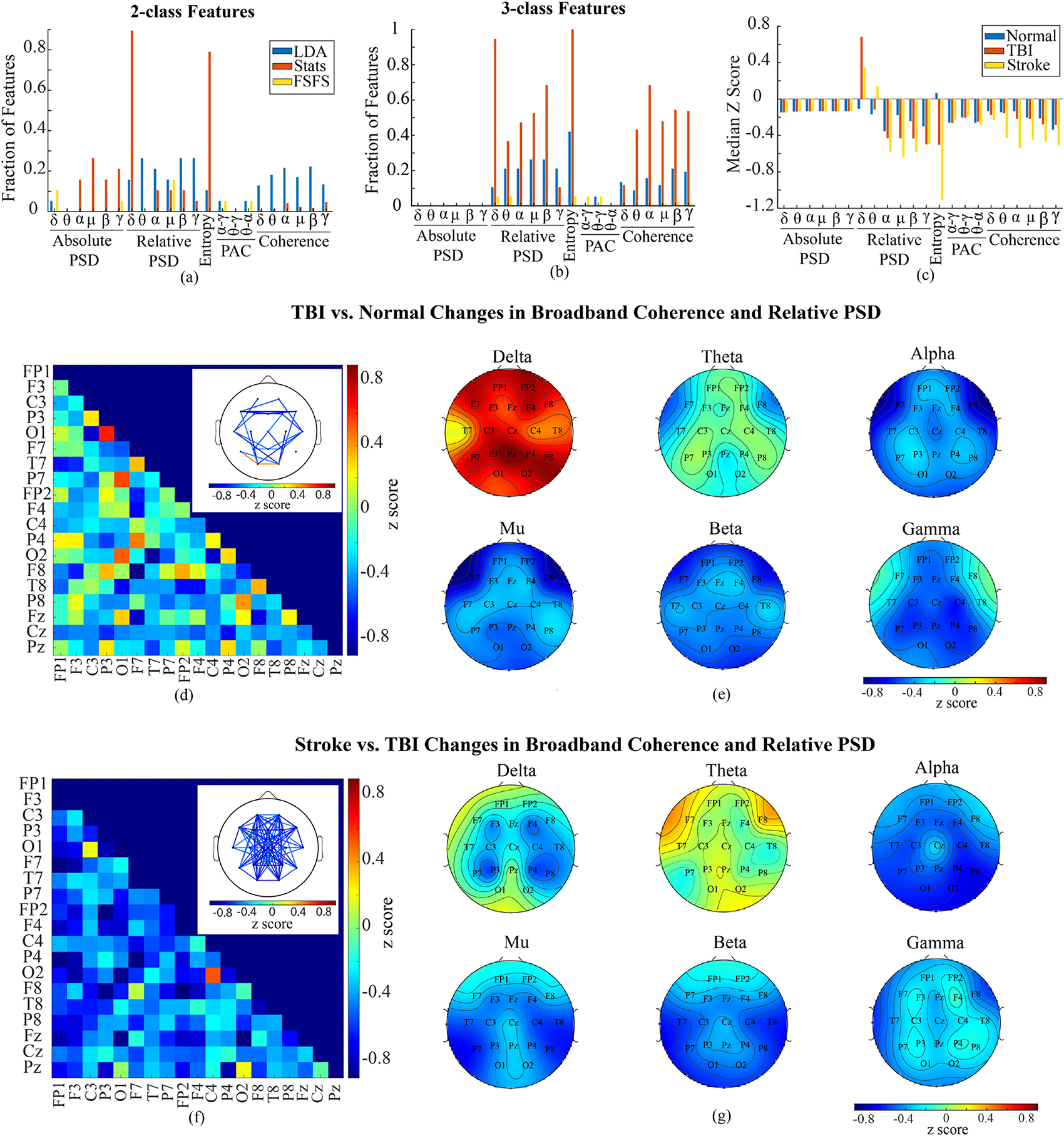
Changes in clean EEG features. (a) shows the fraction of features selected by statistics, LDA, and FSFS out of total number of features in each type of features (i.e., 171 coherence and 19 relative PSD features in each frequency band) without consideration of channels for 2-class classification. (b) shows the fraction of selected features for 3-class classification. (c) shows the median z score for each type of features in normal, TBI and stroke subjects respectively. (d) shows the broadband coherence change from normal to TBI. Main panel shows the median z score of coherence coefficients of all channel pairs. Inset demonstrates the channel pairs with median z score higher than 0.5 or lower than −0.5. (e) shows the topographic map of relative PSD based on z scores. (f) indicates the z score of stroke broadband coherence to TBI. Inset shows the channel pairs with median z score higher than 0.5 or lower than −0.5. (g) shows the topographic map of relative PSD z score of stroke subjects to TBI. (LDA: linear discriminant analysis, FSFS: forward sequential feature selection, Stats: statistics, PAC: phase-amplitude coupling, PSD: power spectral density).

As models built upon features selected by LDA, FSFS, or statistics performed best in both two- and three-class classification, we investigated the composition of these qEEG features and compared them between normal, TBI, and Stroke subjects. Fig. 6 and Sup. Fig. 12 show results obtained from clean and raw EEG respectively. When looking at the fraction of features selected in each qEEG category, most features selected by LDA, FSFS and statistics were PSD and coherence for both two- and three-class classification and both clean and raw EEG (Fig. 6a and 6b, Sup. Fig. 12a and 12b).

To compare between normal, TBI and stroke subjects, we calculated the z score to the standard deviation of normal subjects. Fig. 6c and Sup. Fig. 12c show the median z score (with normal baseline) for each type of feature for all three cohorts. It appears that relative PSD, as well as coherence, had biggest difference in median z scores between the three groups. In addition, in clean EEG data, TBI and stroke patients had significantly reduced entropy compared to normal control.

To further understand the change in coherence, we studied the coherence between all channel pairs between normal and TBI subjects in each frequency band. Though 20–40% of all channel pairs had significant changes across each frequency band compared to normal subjects, the change in coherence was more channel pair dependent rather than frequency band dependent (data not shown), which means the same channel pair often shows the same trend of change across all frequency bands. Therefore, we analyzed the broadband (1–40 Hz) coherence change in TBI for each pair of channels from normal subjects, which is plotted in Fig. 6d and Sup, Fig. 12d. We observed an overall reduction in broadband coherence when only channel pairs with |z| scores higher than 0.5 were analyzed (Fig. 6d inset and Sup. Fig. 12d inset). Reduction in inter-hemisphere coherence was observed across the frontal lobe and between temporal and occipital regions. Reduction in intra-hemisphere coherence was detected between ipsilateral frontal and temporal regions, as well as between frontal and occipital regions. Increase in intra-hemisphere coherence was found between ipsilateral parietal and occipital lobes.

To look at coherence changes in stroke patients from that of TBI patients, we found that 56–72% of all channel pairs had significant changes across all frequency bands (data not shown), and then compared each channel pair as above but with median z-scored data using TBI as a baseline (Fig. 6f and Sup. Fig. 12f). In general, there was a global decrease in most channels with the notable exception of increases between the occipital and ipsilateral central regions (Fig. 6f inset and Sup. Fig. 12f inset).

In addition to coherence, we plotted the topographic maps of relative power change in each frequency band in TBI subjects and stroke subjects, aiming to understand the spatial pattern (Fig. 6e and 6f and Sup. Fig. 12e and 12f). TBI patients showed remarkable increase in relative delta power at parietal and frontal regions, and reduction in alpha and mu power at bilateral fronto-temporal regions compared to normal subjects (Fig. 6e and Sup. Fig. 12e). Stroke patients, when compared to TBI patients, showed decreases in alpha, mu, beta, and gamma frequencies, as well as increases in theta power at fronto-temporal region and decreases in delta power around the bilateral central regions (Fig. 6f and Sup. Fig. 12f).

These analyses reveal complex changes in qEEG features between TBI, stroke and normal subjects, particularly in coherence and relative PSD. In both TBI and stroke patients, coherence showed a global reduction, and relative PSD demonstrated a global increase in low frequency delta frequency band and decrease in high frequency bands. In addition, fronto-temporal and parietal regions appear to have the most remarkable changes in both coherence and relative PSD. In stroke subjects, we saw noticeably lower relative PSD at higher frequencies and higher theta power.

## IV. Conclusion

This study demonstrates that ML models built upon qEEG features and demographic information extracted from existing public databases could distinguish between TBI and normal patients with up to 0.94 accuracy and 0.94 sensitivity in CV and 0.76 accuracy and 0.80 sensitivity in IV. With the addition of a cohort of stroke patients, these models were able to outperform a theoretical model that could only detect changes between normal and abnormal EEGs. In fact, further investigation into the best three-class models showed it distinguished stroke with the highest precision. Feature selection method appears to play the most important role in the performance of models. Our study shows LDA feature selection method outperformed all other methods, reflected by the observation that best performing models in CV and IV for both two- and three-class classification were predominantly based on features selected by LDA (Fig. 5). In diagnosing an independent subject group, SVM with polynomial kernels and coarse KNN performed better than others; while in 10-fold CV, SVM linear or polynomial kernels performed better. In general, including demographic information in the input feature can significantly increase the performance of models, but to a limited degree. Interestingly, models from raw EEG data had a comparable performance with those from clean EEG when just comparing between normal and TBI cohorts. However, when comparing between all three cohorts, clean EEGs performed much better. In line with prior qEEG study on TBI patients, coherence and relative spectral density were two major parameters changed from normal to TBI. Coherence change varied among channel pairs with reduction more predominant. Relative PSD demonstrated a global increase in low frequency delta power and decrease in higher frequency (alpha, mu, beta, and gamma) power. These results suggest EEG ML can potentially be used in the detection or monitoring of TBI in clinic.

## V. Discussion

### A. Use of Temple University Hospital EEG Corpus for TBI and Stroke Research

Temple EEG Corpus is a major, publicly available clinical EEG database [24]. With the advancement of data analysis tools, this database provides an excellent platform for investigators to explore the potential of EEG signals in neurological applications beyond seizure and sleep disorders. In this study, we extracted patients with a record of TBI, those with record of stroke, and those whose EEG was considered normal by clinicians. Demographic distribution (age and sex) of TBI group extracted from the database (Table I) aligns well with that reported previously [25], suggesting that the Temple database represents the general TBI population.

In the Stroke group, the specific type of stroke that had occurred was not always well documented, leaving that group heterogenous in that nature. Though this is a limitation, we hope a large enough sample can either average or dilute any erroneous results.

Ideally, a more homogenous patient population can potentially increase the accuracy in biomarker research. If the database can include the time of onset, the number, severity, and cause of injury, as well as any other available medical record, i.e., neurocognitive test, imaging results, etc., it would be more helpful for the investigation of EEG signals for prognosis and monitoring of the injury, and for the identification of correlations between EEG signals and cognitive function or structural changes. However, the caveats of heterogeneity of TBI patients in the Temple database would not disvalue its importance in the exploration of EEG biomarkers, particularly for incorporating novel analysis methods, i.e., machine/deep learning. EEG signals that can be subjected to multiple quantitative temporal and spectral analyses across multiple channels generate feature sets that are well suited for ML applications. The large number of qEEG features makes a large sample size necessary to develop a reliable classifier, which is difficult to achieve through a single clinical study. Databases like the Temple EEG Corpus become particularly useful in storing and sharing data for integration and re-analysis. In addition to TBI and stroke, we believe such a database can be further used to re-examine the potential of EEG for distinguishing and characterizing different neurological disorders, as the specificity of EEG for different types of neurological disorders is still questionable, which has long prevented its widespread adoption in clinical practice. Our study provides a preliminary evidence on that by demonstrating that ML algorithm can yield high accuracy to separate TBI and stroke patients.

Repository data, while highly desirable for big data and machine learning applications due to its sheer size, is difficult to manage in cases where documentation and metadata formatting is inconsistent. Management tools often require customization on the part of the user, which cause difficulties in open source sharing of both data and algorithms. Medical big data research especially, which aims to uncover relationships and distinctions between various populations, will greatly benefit from the continued efforts to normalize data collection and reporting procedures. In this study, differences in record formatting, gaps in information recorded, and unclear diagnostic outcomes were among the limiting factors in textual analysis, which in turn limits the potential information pool for processing.

### B. EEG Machine Learning and TBI

With the development of advanced analytical techniques and improvement in computational capability, machine/deep learning has been under intense investigation for implementation in multiple neurological fields, including mind decoding in brain-computer interface [26], identification of sleep-wake stages [27], prediction of seizures [28], and prognosis of stroke [29]. Thatcher pioneered utilizing machine learning in the TBI field by applying discriminant analysis of multivariant qEEG features to classify TBI patients and differentiate severe TBI patients from the mild [30], [31]. With 20 EEG features, he achieved an accuracy of >90% in cross-validation and IV. Thornton further tested using 31 high-frequency EEG features to distinguish mild TBI subjects and got an accuracy of about 87% [32]. Recent discriminative index developed from a large sample size and more homogeneous subject population was reported to have a >95% sensitivity to predict positive CT finding in acute TBI [16], and may perform better in monitoring functional recovery from a TBI compared to other clinical outcomes [33]. Furthermore, a multimodal study found that algorithms incorporating EEG signals into symptom questionnaires can increase the accuracy by 10% [34].

Due to the inherent flexibility and wide array of potential algorithmic combinations, as well as the constant advances being made in the field, it is reasonable to make the assumption that newer, more complex models would improve classification results. Here, we evaluated several common ML techniques with a range of parameters in order to determine potential utility in TBI classification tasks. In our study, we achieved an accuracy up to 94% in CV and 76% in IV. Six of the top 12 performing models in 10-fold CV (with the other 6 using BSFS based on LDA features) and 6 out of 18 best performing models in IV used LDA as the feature selection method. Since LDA can function independently as a binary classifier and has been most often used in prior EEG TBI reports, it is well suited for separating the classes examined here. When used as a multiclass classifier, a one vs one coding strategy was used, where only two classes were looked at each iteration. The BSFS selection method was unique in that it started with the features selected from LDA, but removed any additional features it could, leaving us with a smaller feature set but comparable performance in 10-fold CV. However, the models trained with features from BSFS preformed significantly worse in IV. This is mostly likely due to overfitting on the original training data caused by optimization on an already optimized dataset.

The LDA and BSFS methods selected 178–224 EEG features, PCA selected just over 130 features, conventional statistics selected 80–98 features, and FSFS selected 12 or fewer features. This is surprising since the other best performing models in the IV used features selected from the smallest number of features selected by FSFS or those showing significant difference statistical differences. Overall, the features selected by LDA and conventional statistics shared up to 28 raw features, while only 2–4 raw FSFS features were found in both. Though the number of features from LDA and statistics appeared to be high relative to the number of subjects, we implemented multiple folds of validation to reduce the impact of overfitting, including 10-fold CV, randomizing the labeling, and IV. In addition, SVM was used for its good performance in handling high-dimensional data. Indeed, 21 out of the 30 best performing models were SVM models, with only nine trained with KNN kernels in two-class classification. And all best performers in three-class classification are SVM models. In future work, other methods such as LASSO and convolutional neural network (CNN) may be implemented to further improve the dimensional reduction and possibly the classification results. CNN has been shown to have superior performance in neurological applications compared to conventional algorithms and may provide higher sensitivity and specificity [35]–[38].

Although we had moderate success with CV accuracy, it is troubling that there was 18 point drop in IV accuracy. In general, a drop in performance between CV and IV is indicative of overfitting during training. A common way this is addressed is by increasing the sample size for training sets, which was not feasible here based on the inclusion/exclusion criteria and the data available in Temple EEG Corpus. A larger sample size for model training, with consistent data labels, could address this limitation. Alternatively, this study used randomly labeled data to generate a baseline performance margin for each model. IV classification consistently outperformed classification of subjects randomly assigned to a cohort, showing that, while overfit to the training set used for CV, the overall models were still able to generalize to unseen data. Future work will focus on improving IV accuracy, either through larger training sets or more advanced algorithms (deep learning, ensemble methods, etc.).

As suspected, most models performed better than conventional statistics. The exception to this was the surprisingly poor results of the PCA results (Fig. 5). Since PCA relies on a linear transformation, it is possible that the features are better suited for nonlinear transformations. However, this is contradictory to the success found with the linear LDA SVM model, so further investigation is needed. The other high performing models (Sup. Table I and II) consist of mostly polynomial SVMs. This is perhaps due to their versatility to this set of heterogenous set of data that could not be captured in the KNN models.

It also needs to be noted that almost all models published for TBI classification utilized supervised learning. However, one of the challenges in the detection and monitoring of TBI is the lack of an early and sensitive outcome measure. This restrains the performance of classifiers within our current knowledge breadth. McCrea et al. [33] reported that an EEG-based algorithm could potentially be more sensitive than conventional neurocognitive assessment in monitoring the recovery from TBI. Our own study in mice also suggests EEG changes can be observed without an apparent neuroinflammatory reaction [12]. Therefore, in the future, an unsupervised approach can be explored to mitigate this limitation.

Our study also explored the effect of demographic information and artifact removal on the performance of models. Due to the significant difference in the rate of TBI between males and females, including demographic information can slightly increase the accuracy of models developed with the same algorithm. However, its effect on the model performance is less than input features, algorithms, and kernels. Interestingly, models developed from features calculated from raw EEG data demonstrated comparable performance with those trained with clean EEG features in two-class classification. However, artifact-removal significantly increased the performance of three-class models. We speculated that some information embedded within artifact, i.e., eye movement, could be different between TBI and normal subjects, explaining this discrepancy. A further investigation into the IClabels removed in our work indeed show a significant difference in the number of eye movement artifacts between normal and TBI groups (Sup. Fig. 13). Moreover, a significant difference in the number of muscle and eye movement artifacts was revealed between TBI and stroke patients. In addition, it appears that artifact removal noticeably changed the coherence difference between stroke and TBI subjects. This may partially explain why artifact removal did not affect the two-class model performance but increased the performance of three-class models.

### C. QEEG Differences Between Normal, TBI and Stroke

Though identifying qEEG biomarkers was not the primary goal of this study, understanding changes can help us compare results from the database with prior reports, and determine the features that can significantly contribute to a well-performing model. LDA, statistics, and FSFS selected a remarkable fraction of features from coherence and relative PSD for both two- and three-class classification (Fig. 6a). Stroke and TBI differ in the cause of brain injury (internal vs. external), whereas share some pathological processes including the primary cranial cell death and blood-brain-barrier disruption followed by secondary neuroimmune responses triggered by cytokines. Due to the similarities in pathophysiology and associated functional deficits between these two conditions, specificity of EEG in distinguishing these two conditions is always questionable. Indeed, our study suggests similar trend of qEEG changes in coherence and relative PSD between the two groups with changes in stroke patients more prominent. However, ML models reasonably separated these two groups, suggesting advanced analytics can potentially be more sensitive to identify differences compared to conventional statistics.

In literature report, the trend of change in coherence related to TBI and stroke varies, however, the most reported was the reduction in global or inter-hemisphere coherence [6], [8]. Other reports suggest the change can be pathway-specific [30], [39]. Our study seems to support both. The grouped analysis (Fig. 6d inset) shows a symmetric pattern between two hemispheres in TBI patients. Though this does not mean the same patient would have symmetric changes, it suggests the same pathway in both hemispheres are equally susceptible to the same change. When comparing stroke to TBI, stroke patients showed a further reduction in global coherence, reflecting a more severe interruption of inter-neuronal communication, which is consistent with prior report [40].

Unlike diverse findings in coherence change after TBI and stroke, reports in PSD alteration were more consistent with an increase in lower frequency bands (delta and theta) and a reduction in higher frequency bands (alpha, beta and gamma) [41], [10], [6], [42]. Our study revealed the same trend of change in relative PSD as shown in Fig. 6e and 6g, however, stroke had additional increase in theta power.

In addition to coherence and PSD, LDA extracted several entropy features in raw EEG, and a large number of entropy features were found significantly different from groups in clean data. Different metrics for entropy have been employed to identify EEG biomarkers of TBI and stroke. A decrease at the acute phase of injury followed by a recovery was mostly reported in both animal and human studies for TBI [12], [43], [44]. Though TBI subjects in the Temple database were diverse, which may include chronic injury with entropy recovered and those with local injury [45], an overall reduction was remarkable in clean EEG. For stroke patient, an increase in sample entropy was reported previously [40], this is controversial to what we found in clean EEG data that a significant reduction was revealed in stroke group. Further studies on entropy changes in stroke and TBI patients are needed to determine its post-stroke and post-TBI alterations. The reason that clean data demonstrated entropy change but not raw EEG may be because the cleaning process utilizes features associated with entropy. Since signal noise increases overall variance, removing artifacts through cleaning methods reduces noise and therefore should alter extracted entropy features. Without artifact signals (i.e., eye-movement) buffering entropy values, changes in these features can be more easily attributed to the classes.

While further work is necessary to develop clinically applicable spectral feature biomarkers and accompanying diagnostic models for TBI, research indicates that EEG data provides a measure of separability between normal and TBI subjects, and with potential to separate TBI from other neurological conditions, i.e., stroke. Other non-invasive, portable modalities may be combined with EEG to enhance the available information within the feature set for these types of analyses. Future work will investigate the biological basis of the relationship between selected features and TBI pathology, as well as algorithmic improvements to modeling neurological disorders, for classification purposes.

## Supplementary Material

Supplementary Material

## Figures and Tables

**TABLE I:** Demographic Information of Normal, TBI and Stroke Subjects. P-Values Calculated With Chi-Square (Sex) or One Way ANOVA and Post-Hoc Tukey Test (Age and Medication). No Statistics Were Done on Age Range.

**Training Dataset**						
	**Normal (n=79)**	**TBI (n=98)**	**Stroke (n=115)**	**p value (normal vs. TBI)**	**p value (normal vs. stroke)**	**p value (TBI vs. stroke)**
Sex (M/F/unknown)	29/47/3	74/20/4	62/47/6	p<0.000l	p<0.000l	p<0.000l
Age (n=unknown)	47.6±18.7 (n=2)	42.7±16.7 (n=l)	59.7±9.7 (n=l)	p=0.08	p<0.000l	p<0.000l
Age range	2.9–85	12–80	33–74			
Medication	1.4±1.9	1.4±2.1	1.5±2	p=0.96	p=0.89	p=0.98
**Independent Validation Dataset**						
	**Normal (n=26)**	**TBI (n=44)**	**Stroke (n=50)**	**p value (normal vs. TBI)**	**p value (normal vs. stroke)**	**p value (TBI vs. stroke)**
Sex (M/F/unknown)	8/18/0	34/8/2	26/23/1	p<0.001	p<0.001	p<0.001
Age (n=unknown)	53.1±18.4	41.8+16.1	59.3+12.1	p<0.01	p=0.21	p<0.000l
Age range	19–81	18–79	5–73			
Medication	0.6±0.9	1.5±2	1.1±2	p=0.08	p=0.52	p=0.41
