# Supplementary material for: Evaluating Performance of EEG Data-Driven Machine Learning for Traumatic Brain Injury Classification
Source: IEEE Trans Biomed Eng. Author manuscript; Available in PMC 2022 Sep 27. (PMC9513823; doi:10.1109/TBME.2021.3062502)
Supplement: Supplementary Material [file NIHMS1833746-supplement-Supplementary_Material.pdf]

Supplemental Materials

Evaluating Performance of EEG Data-Driven Machine Learning for Traumatic Brain Injury Classification

Nicolas Vivaldi[*], Michael Caiola[*], Krystyna Solarana, and Meijun Ye

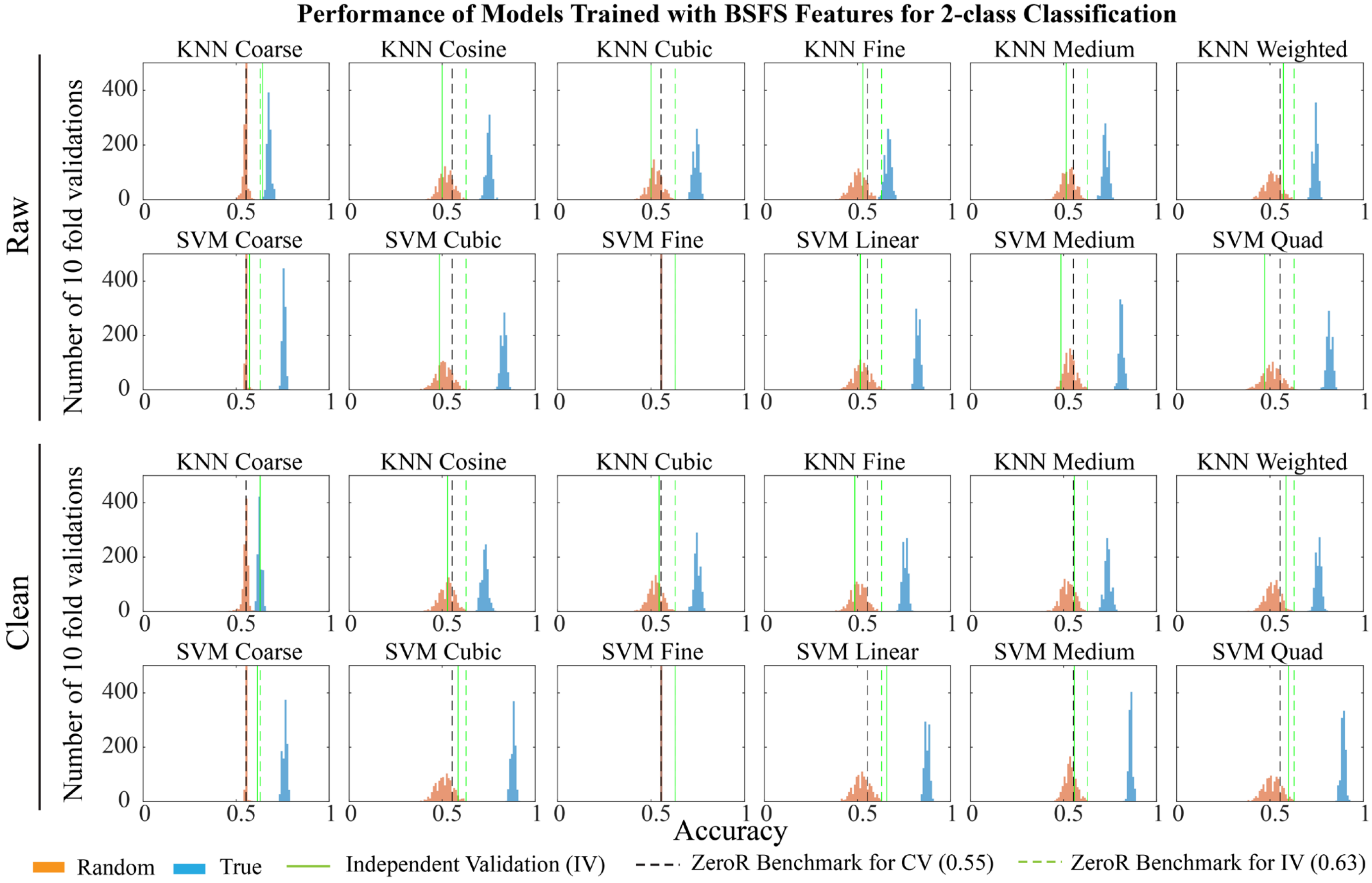

Supplemental Figure 1. Performance of models trained with features selected by backward sequential feature selection (BSFS) for 2-class classification. All true-label distributions are significantly different from randomly label distributions (two-sample K-S test, $p<10^{-10}$) except for both raw and clean SVM Fine Gaussian. Green line indicates the accuracy of models to classify the independent dataset. Black and green dotted lines show ZeroR benchmarks for cross-validation (CV) and independent validation (IV) respectively. (SVM: support vector machine, KNN: K-nearest neighbors).

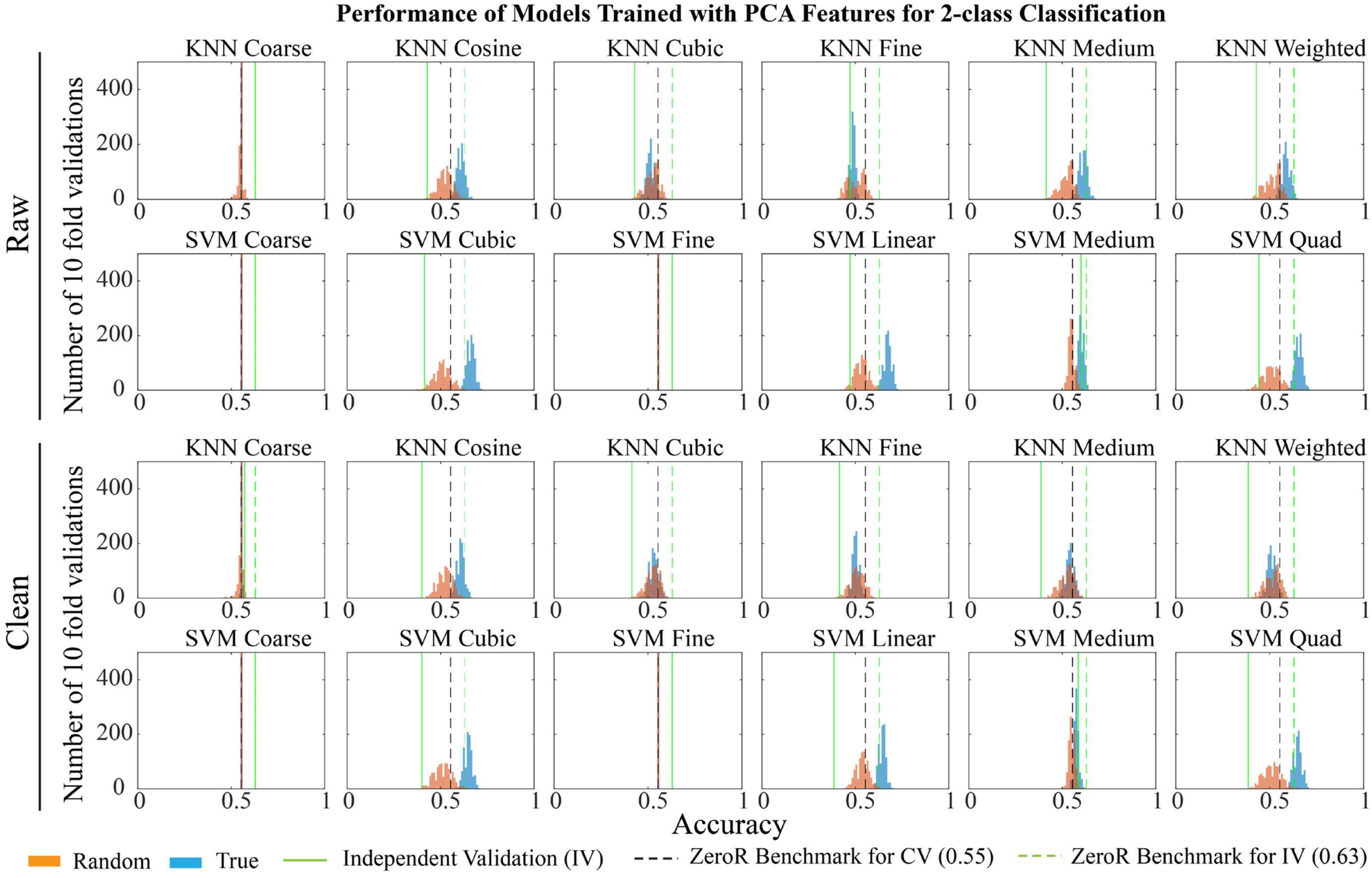

Supplemental Figure 2. Performance of models trained with Principal Component (PC) features that account for ≥ 95% of the total variation for 2-class classification. All true-label distributions are significantly different from randomly label distributions (two-sample K-S test, $p<10^{-10}$) except for both raw and clean support vector machine (SVM) Coarse and Fine Gaussian and clean K-nearest neighbors (KNN) Cubic Distance. Green line indicates the accuracy of models to classify the independent dataset. Black and green dotted lines show ZeroR benchmarks for cross-validation (CV) and independent validation (IV) respectively. (SVM: support vector machine, KNN: K-nearest neighbors).

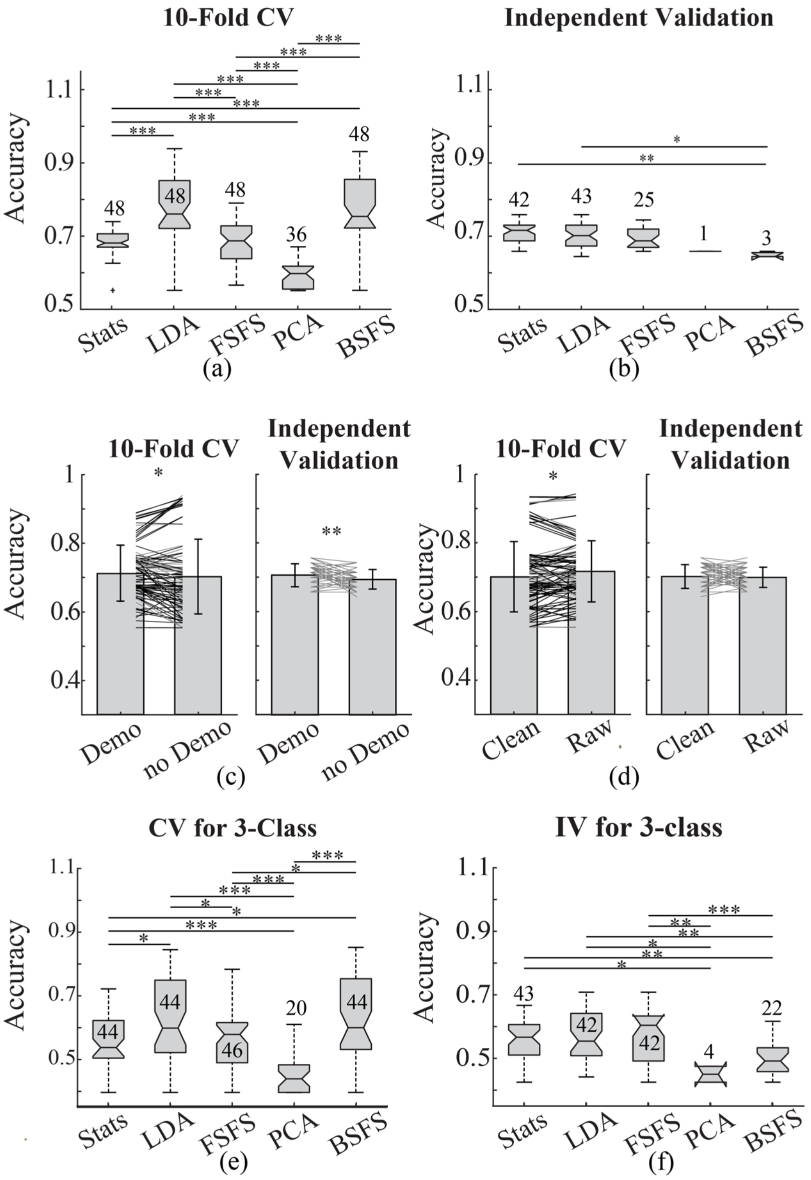

Supplemental Figure 3. Comparison of performance of models above ZeroR for 2-class and 3-class classification. **(a) and (b)** show boxplots of accuracy of models trained with features selected by different methods excluding those with below ZeroR accuracy for 2-class classification. Only few models trained with PCA and BSFS features demonstrated an accuracy above ZeroR. **(c)** compares the accuracy of models trained with input features including demographic information and those without demographic information (Demo: demographic). The majority models with demographic inputs appear to perform better than their counterparts. **(d)** compares the performance of models trained with features generated from artifact removed clean EEG data versus those from raw EEG. Each line (c) and (d) represents each algorithm. Dark lines in the left panels of (c) and (d) indicate significant difference in two sample K-S test at 10^{-10} SL. **(e)** and **(f)** demonstrate boxplots of accuracy of models above ZeroR for 3-class classification. (* $p < 0.05$, ** $p < 0.01$, *** $p < 0.001$, One way ANOVA and post-hoc Tukey test in (a) and (b), Signed-rank test in (c) to (f)). (CV: cross-validation, IV: independent validation, Stats: statistics, LDA: linear discriminant analysis, FSFS: forward sequential feature selection, PCA: principal component analysis, BSFS: backwards sequential feature selection)

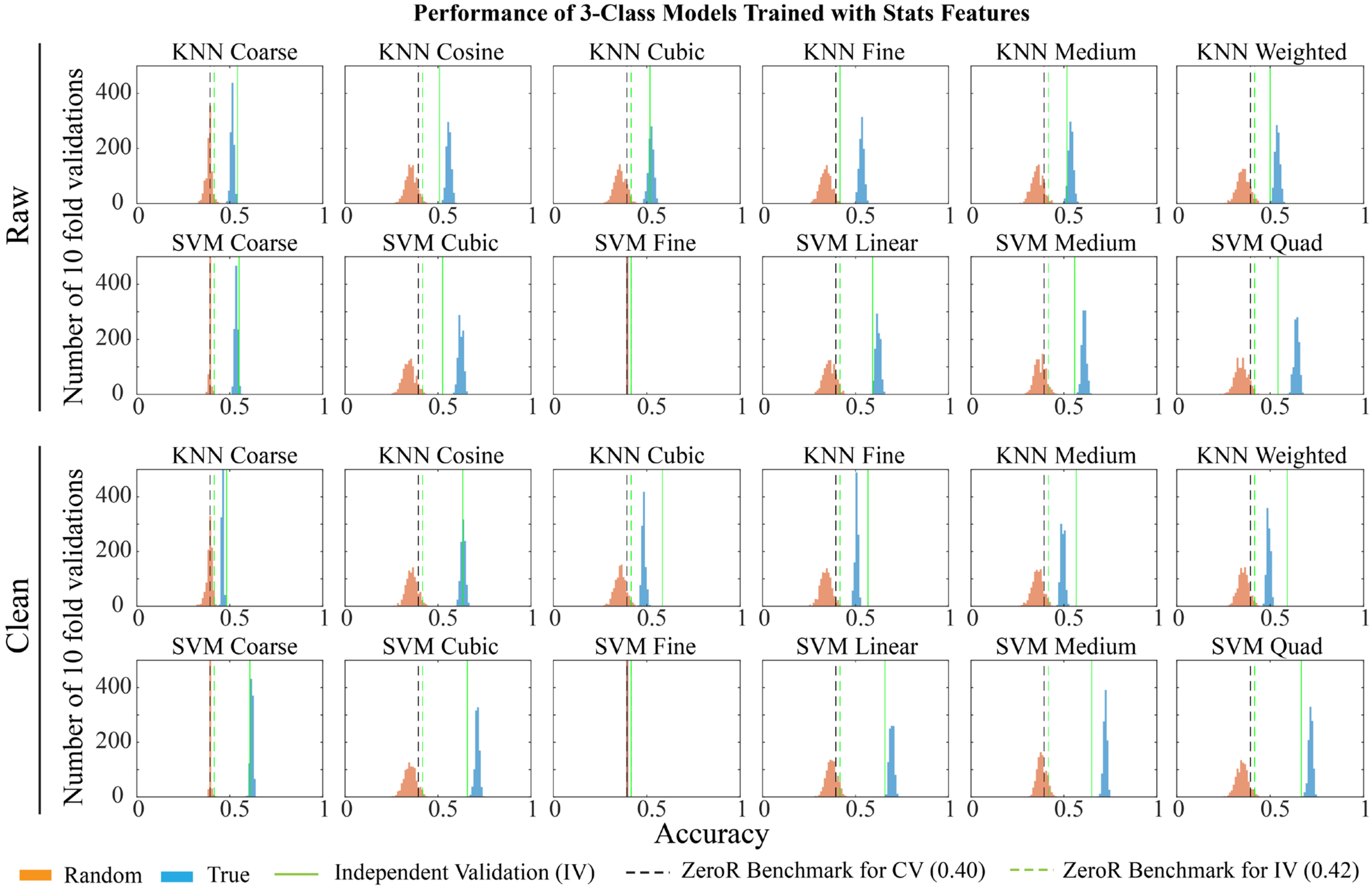

Supplemental Figure 4. Performance of models trained for 3-class classification with features selected by conventional statistics (see Methods). Green line indicates the accuracy of models to classify the independent dataset. Black and green dotted lines show ZeroR benchmarks for cross-validation (CV) and independent validation (IV) respectively. (SVM: support vector machine, KNN: K-nearest neighbors).

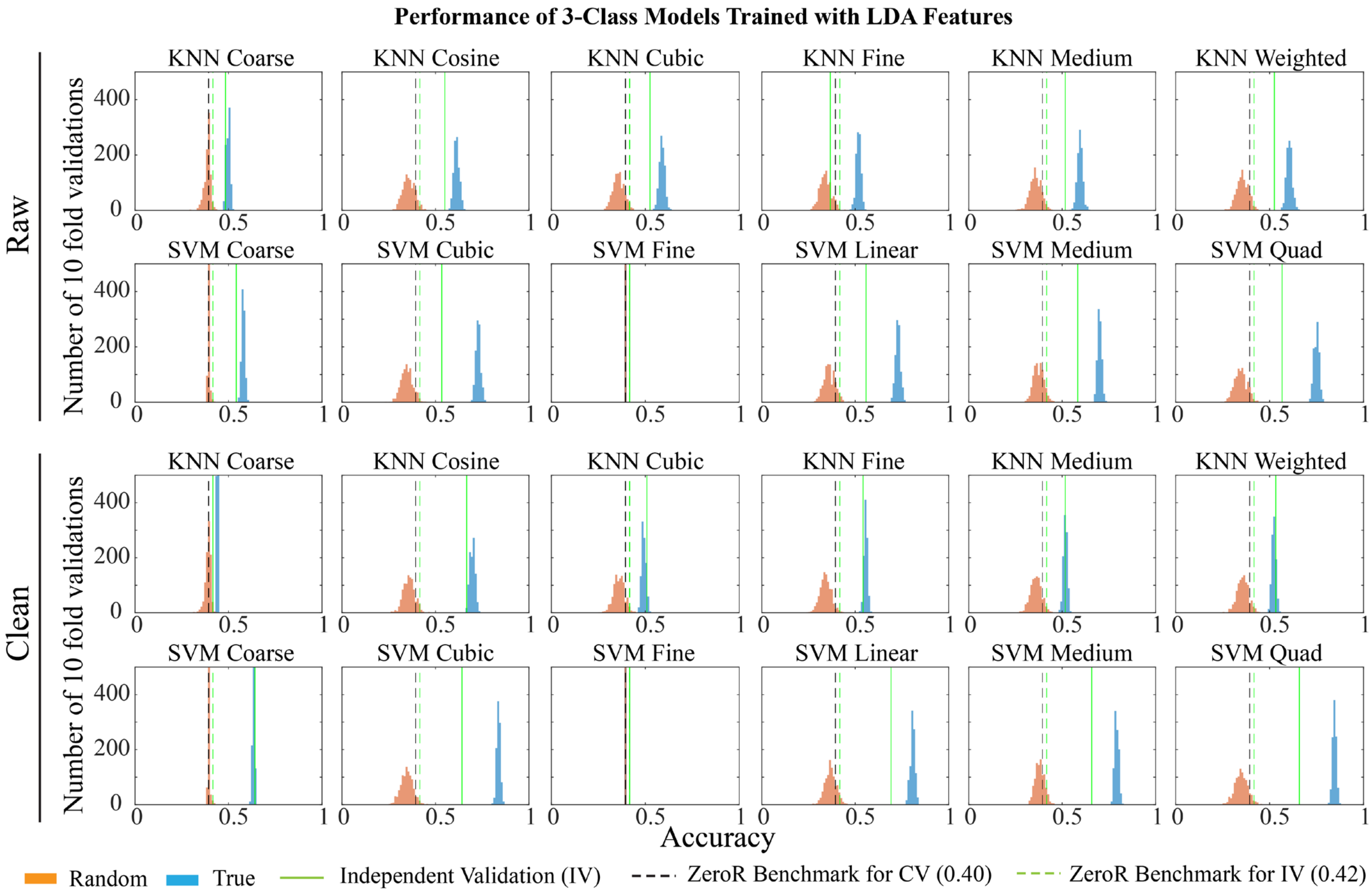

Supplemental Figure 5. Performance of models trained for 3-class classification with features selected by Linear Discriminative Analysis (LDA) (see Methods). Green line indicates the accuracy of models to classify the independent dataset. Black and green dotted lines show ZeroR benchmarks for cross-validation (CV) and independent validation (IV) respectively. (SVM: support vector machine, KNN: K-nearest neighbors).

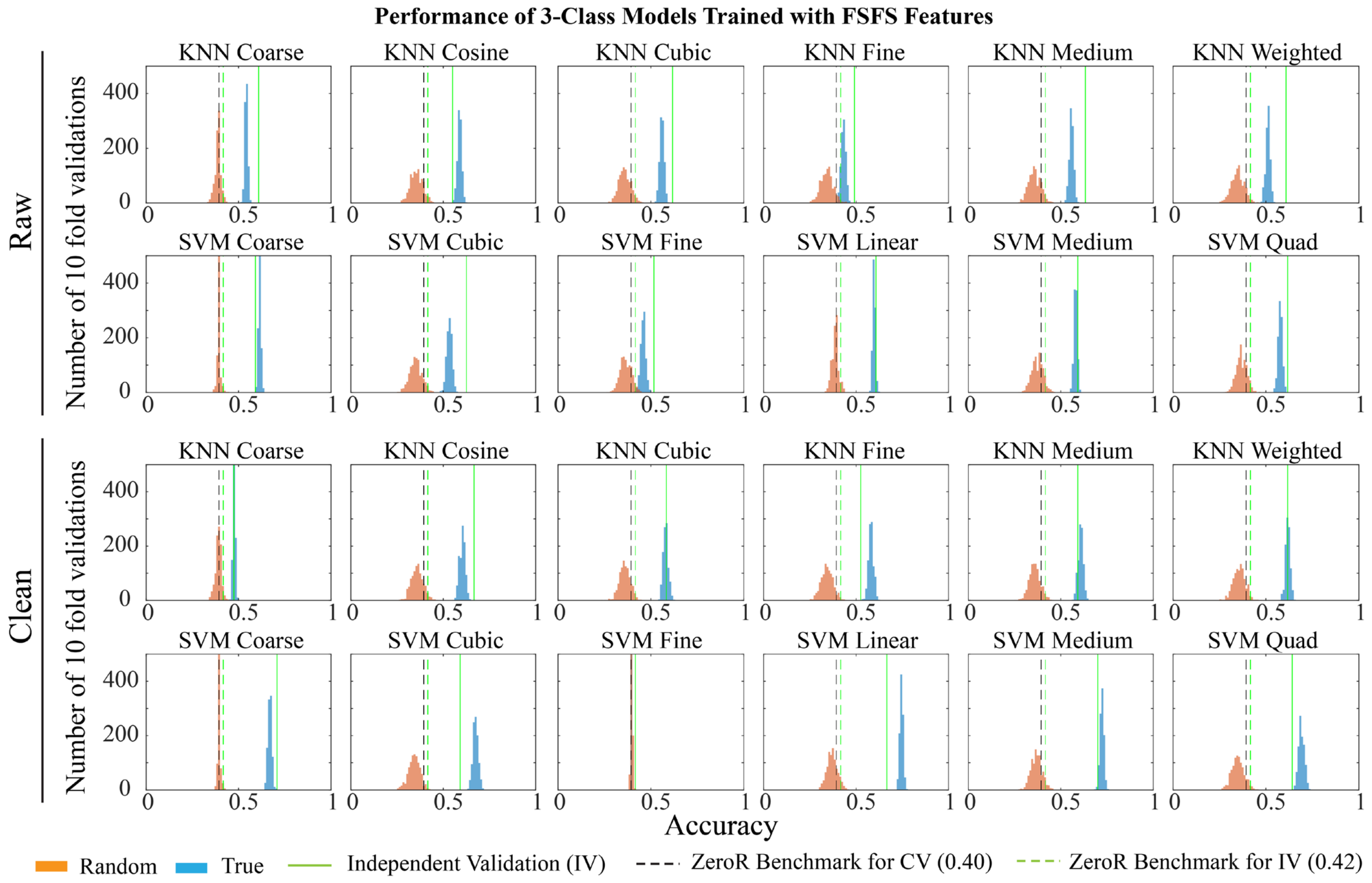

Supplemental Figure 6. Performance of models trained for 3-class classification with features selected by Forward Sequential Feature Selection (FSFS) (see Methods). Green line indicates the accuracy of models to classify the independent dataset. Black and green dotted lines show ZeroR benchmarks for cross-validation (CV) and independent validation (IV) respectively. (SVM: support vector machine, KNN: K-nearest neighbors).

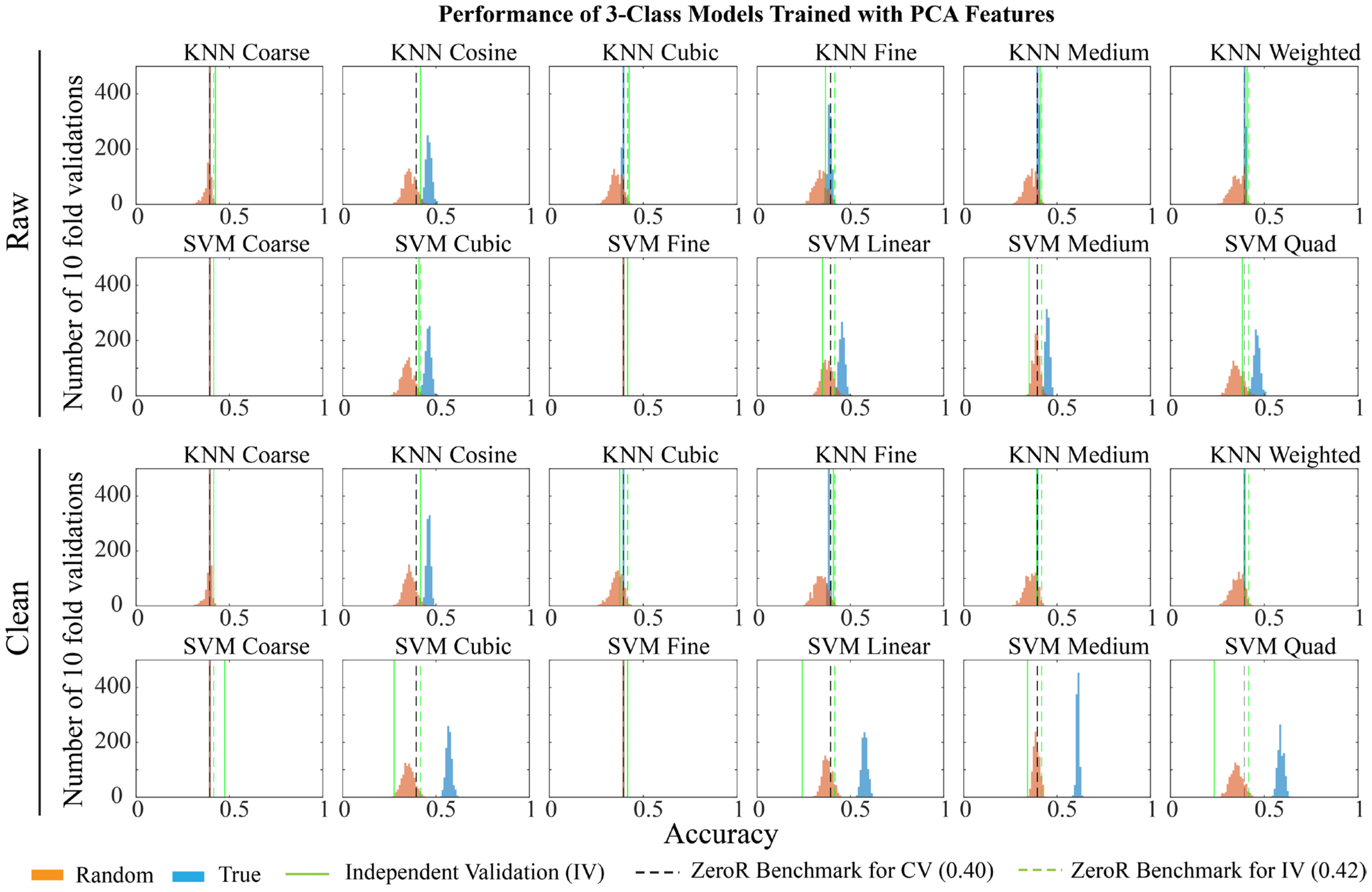

Supplemental Figure 7. Performance of models trained with Principal Component (PC) features that account for ≥ 95% of the total variation for 3-class classification. Green line indicates the accuracy of models to classify the independent dataset. Black and green dotted lines show ZeroR benchmarks for cross-validation (CV) and independent validation (IV) respectively. (SVM: support vector machine, KNN: K-nearest neighbors).

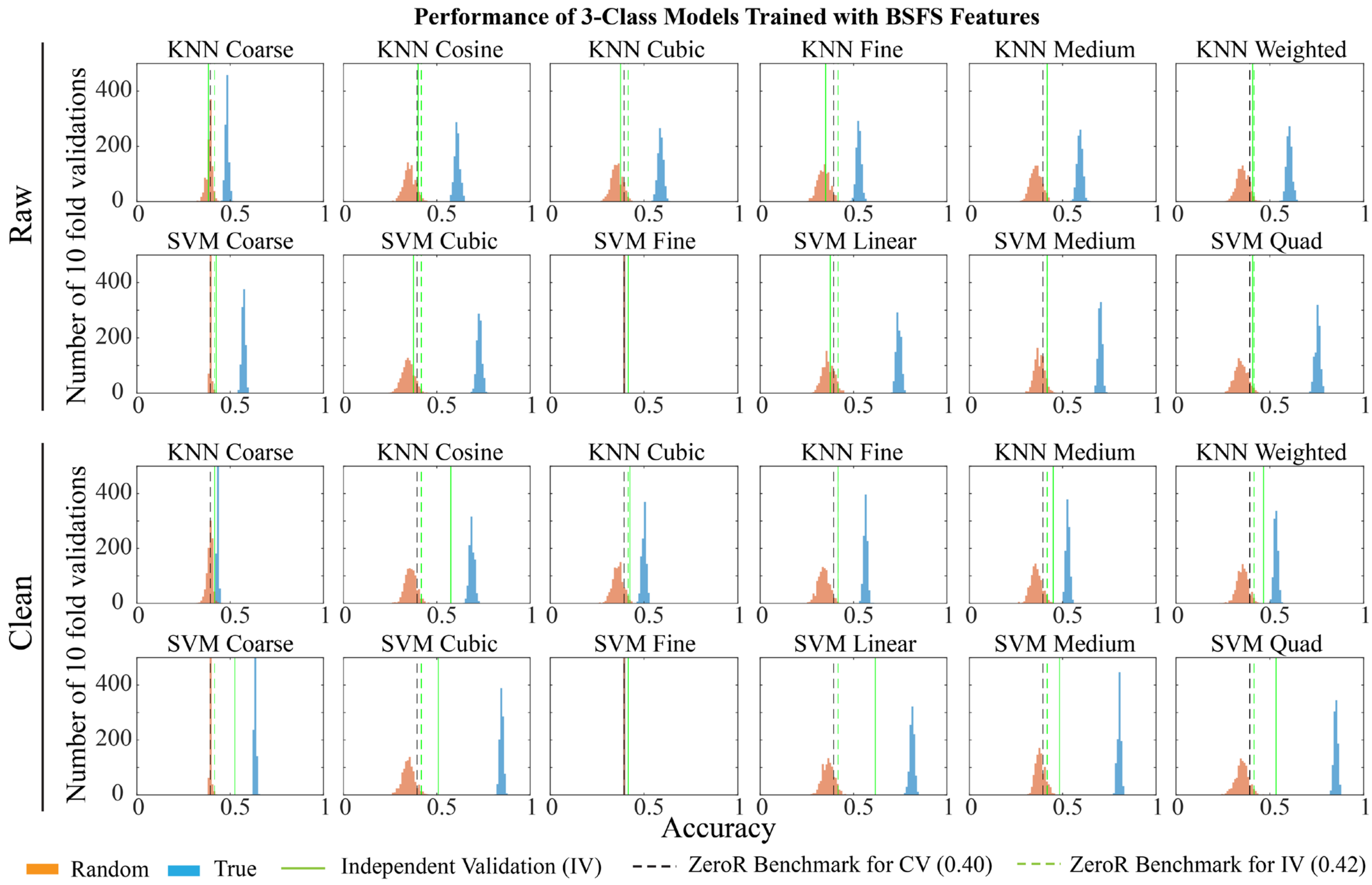

Supplemental Figure 8. Performance of models trained with features selected by BSFS for 3-class classification. Green line indicates the accuracy of models to classify the independent dataset. Black and green dotted lines show ZeroR benchmarks for cross-validation (CV) and independent validation (IV) respectively. (SVM: support vector machine, KNN: K-nearest neighbors).

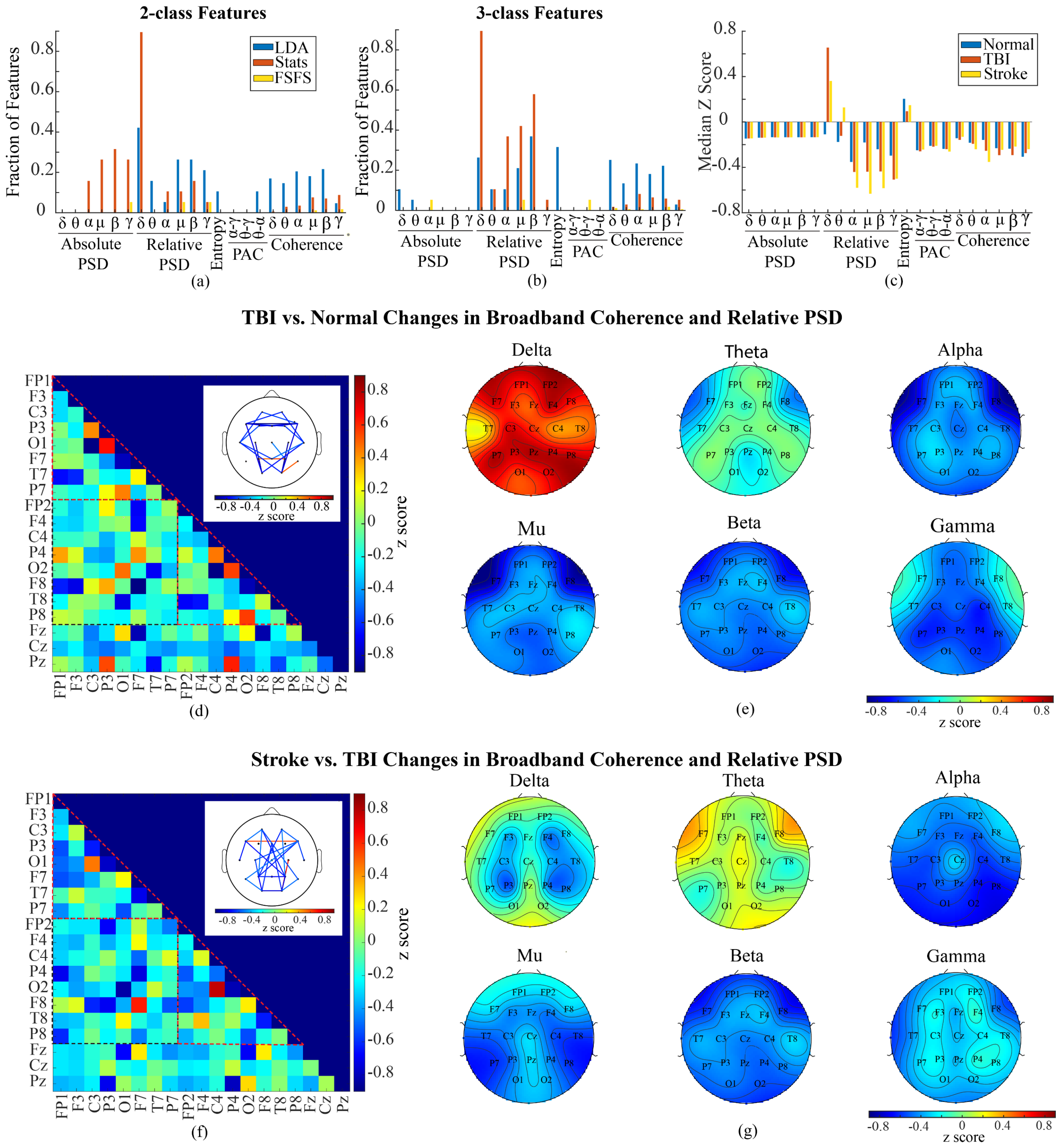

Supplemental Figure 9. Changes in raw EEG features. (a) shows the fraction of features selected by statistics, LDA, and FSFS out of total number of features in each type of features (i.e., 171 coherence and 19 relative PSD features in each frequency band) without consideration of channels for 2-class classification. **(b)** shows the fraction of selected features for 3-class classification. **(c)** shows the median z score for each type of features in normal, TBI and stroke subjects respectively. **(d)** shows the broadband coherence change from normal to TBI. Main panel shows the median z score of coherence coefficients of all channel pairs. Black dotted box indicates inter-hemisphere coherence, while red dotted triangle boxes indicate intra-hemisphere coherence. Inset demonstrates the channel pairs

with median z score higher than 0.5 or lower than -0.5. **(e)** shows the topographic map of relative PSD based on z scores. **(f)** indicates the z score of stroke broadband coherence to TBI. Inset shows the channel pairs with median z score higher than 0.5 or lower than -0.5. **(g)** shows the topographic map of relative PSD z score of stroke subjects to TBI. (LDA: linear discriminant analysis, FSFS: forward sequential feature selection, Stats: statistics, PAC: phase-amplitude coupling, PSD: power spectral density).

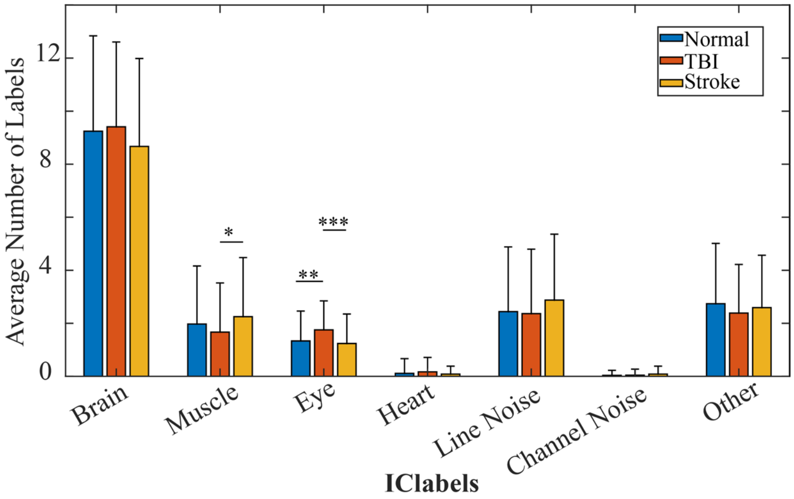

Supplemental Figure 10. Independent Component (IC) of artifact removed from each cohort of patients. (*<0.05, **<0.01, ***<0.001, One way ANOVA and post-hoc Tukey test)
